# Overt and subclinical hypothyroidism during pregnancy: effects on neonatal amino acids, acylcarnitines, and TSH levels

**DOI:** 10.3389/fendo.2026.1872893

**Published:** 2026-09-25

**Authors:** Min Wu, Fei Kong, Shuting Huang, Qiongfang Yao, Mei Ma, Yinglin Zeng, Jinying Luo, Jinfu Zhou

**Affiliations:** 1 Medical Genetic Diagnosis and Therapy Center, Fujian Maternity and Child Hospital College of Clinical Medicine for Obstetrics & Gynecology and Pediatrics, Fujian Medical University, Fuzhou, Fujian, China; 2 Fujian Provincial Key Laboratory for Prenatal Diagnosis and Birth Defect, Fuzhou, Fujian, China; 3 Department of Preventive Medicine, School of Public Health, Fujian Medical University, Fuzhou, Fujian, China; 4 School of Medical Technology and Engineering, Fujian Medical University, Fuzhou, Fujian, China; 5 Department of Laboratory Medicine, Fujian Maternity and Child Health Hospital, College of Clinical Medicine for Obstetrics & Gynecology and Pediatrics, Fujian Medical University, Fuzhou, Fujian, China; 6 Obstetrics and Gynecology Department, Fujian Maternity and Child Hospital College of Clinical Medicine for Obstetrics & Gynecology and Pediatrics, Fujian Medical University, Fuzhou, Fujian, China

**Keywords:** acylcarnitines, amino acids, hypothyroidism during pregnancy, neonatal metabolism, overt hypothyroidism, subclinical hypothyroidism

## Abstract

**Background/objectives:**

Maternal hypothyroidism during pregnancy, including overt and subclinical hypothyroidism, is a common endocrine disorder complicating pregnancy and is associated with increased risks of adverse maternal and fetal outcomes. However, its effects on neonatal metabolism remain inadequately understood. This study aimed to assess the association between hypothyroidism in pregnancy and neonatal amino acid and acylcarnitine levels.

**Methods:**

This retrospective case–control study included 1,987 pregnant women with overt hypothyroidism in pregnancy, 2,023 with subclinical hypothyroidism in pregnancy, and 2,043 euthyroid pregnant women from Fujian Provincial Maternity and Children’s Hospital between 2016 and 2024. Their newborns all underwent newborn screening for 11 amino acids and 31 acylcarnitines using tandem mass spectrometry.

**Results:**

After adjusting for potential confounding factors including gestational age at delivery, birth weight, and neonatal sex, newborns born to mothers with hypothyroidism in pregnancy (overt and subclinical) showed significant differences in the levels of 9 amino acids compared with those born to mothers with normal thyroid function. These amino acids are involved in the arginine-proline metabolism, glycine metabolism, and the urea cycle pathway. Amino acid levels also differed between the two subtypes. In addition, the levels of short-chain, medium-chain, and long-chain acylcarnitines in newborns from the hypothyroidism groups were significantly higher than those in the control group, with several acylcarnitine indicators (C16, C16:1, C16OH, C18:1OH, and C6DC) exhibiting characteristic differences between the two subtypes. The levels of thyroid-stimulating hormone (TSH) in newborns from both hypothyroidism groups were significantly higher than those in the control group, whereas no statistically significant difference in TSH levels was observed between the overt and subclinical hypothyroidism groups.

**Conclusion:**

Our findings revealed a significant association between hypothyroidism in pregnancy (overt and subclinical) and neonatal amino acid and acylcarnitine levels, which were mainly involved in branched-chain amino acid, arginine, proline, and glycine metabolism, as well as the urea cycle and fatty acid oxidation pathways. These results underscore the importance of identifying maternal conditions that correlate with neonatal metabolites, providing a reference for exploring strategies to optimize neonatal nutrition and improving long-term health outcomes.

## 1 Introduction

Maternal hypothyroidism during pregnancy, including overt hypothyroidism and subclinical hypothyroidism, is one of the most common endocrine and metabolic disorders. It is characterized by elevated serum thyroid-stimulating hormone (TSH) levels, accompanied by decreased (in overt hypothyroidism) or normal (in subclinical hypothyroidism) free thyroxine (FT4) levels (1, 2). The global prevalence of hypothyroidism during pregnancy is approximately 4% (including 0.5% overt hypothyroidism and 3.5% subclinical hypothyroidism), with subclinical hypothyroidism accounting for over 85% and thus being the predominant type (1). Moreover, the popularization of prenatal thyroid function screening, changes in iodine nutritional status, and the rising obesity rate indirectly contribute to the increase in its detection rate (3, 4). Thyroid hormones during pregnancy are key regulators of maternal metabolism and are critical for sustaining the normal growth and development of the fetus. Particularly in the first trimester, when the fetal thyroid gland is not yet functionally mature, the development of the fetal nervous system and organs is entirely dependent on the supply of thyroid hormones from the mother (5, 6). Insufficient secretion of maternal thyroid hormones not only increases the risk of adverse pregnancy outcomes such as gestational diabetes mellitus, hypertensive disorders of pregnancy, placental abruption, preterm birth, and low birth weight, but also exerts long-term adverse effects on the fetus (5, 7, 8). This hormonal insufficiency significantly elevates the risk of cognitive developmental delay, intellectual disability in the offspring during childhood, as well as metabolic syndrome and cardiovascular diseases in adulthood (5, 9, 10). This has become a critical issue requiring urgent attention in perinatal healthcare.

Thyroid hormones are core metabolic regulatory hormones that maintain systemic metabolic homeostasis by regulating the synthesis and catabolism of carbohydrates, lipids, and proteins. Abnormalities in their secretion can directly induce systemic metabolic disorders in the mother (11, 12). The fetus’s nutrient supply and metabolic regulation mainly depend on placental material transport. Metabolic imbalances caused by maternal thyroid dysfunction can cross the placental barrier to affect fetal metabolism, leading to disturbances in the metabolic processes of key metabolites such as fatty acids and cholesterol (13, 14). Newborn screening (NBS), which detects amino acid and acylcarnitine levels in newborns’ heel-stick dried blood spots, is not only a core method for screening inborn errors of metabolism but also provides an objective reflection of the fetal intrauterine metabolic status through its detection indicators. These indicators serve as important molecular phenotypic biomarkers for assessing the impact of the intrauterine environment on fetal metabolism (15, 16). As the basic units of protein synthesis, amino acids are involved in multiple physiological processes including fetal nervous system development and cell proliferation and differentiation, while acylcarnitines serve as key carriers for fatty acid β-oxidation, and together they form the core metabolic basis for fetal intrauterine growth and development (17–19). Alterations in fetal amino acid and acylcarnitine profiles induced by maternal metabolic disorders may also serve as important predisposing factors for long-term metabolic and neurodevelopmental abnormalities in offspring via the mechanism of the Developmental Origins of Health and Disease (DOHaD) (20–22).

In recent years, the advancement of NBS technology has expanded its application beyond the screening of congenital diseases; it is also used to assess the impact of various adverse intrauterine exposures on neonatal metabolism. For instance, factors such as gestational hyperglycemia and hypertensive disorders of pregnancy have been confirmed to significantly alter the amino acid and acylcarnitine profiles of newborns (16, 19, 22). Studies have confirmed that hypothyroidism during pregnancy can interfere with maternal protein synthesis and fatty acid oxidation metabolism, downregulate the expression and activity of placental amino acid transporters, and thereby affect the effective supply of amino acids to the fetus *in utero* (23, 24). Meanwhile, studies suggest that hypothyroidism may also indirectly impact the synthesis and transport of fetal acylcarnitines by disrupting fatty acid metabolic pathways (25, 26). However, most relevant studies have focused on single or a few indicators (either amino acids or acylcarnitines) and have not systematically analyzed the comprehensive profiles of amino acids and acylcarnitines in newborns. Furthermore, overt and subclinical hypothyroidism exert significantly different effects on maternal metabolism due to varying degrees of thyroid hormone deficiency (25, 27). However, it remains unclear whether the two subtypes exert subtype-specific effects on fetal intrauterine metabolism and lead to characteristic differences in neonatal amino acid and acylcarnitine profiles.

Therefore, this study aimed to employ the NBS platform to determine the amino acid and acylcarnitine levels in neonates, investigate the effects of overt hypothyroidism and subclinical hypothyroidism during pregnancy on the neonatal metabolic profile, compare the distinct changes in amino acid and acylcarnitine patterns induced by the two subtypes, and explore the underlying mechanisms by which hypothyroidism during pregnancy affects fetal metabolism *in utero*.

## 2 Materials and methods

### 2.1 Study design and population

This retrospective case–control study included 1,987 singleton pregnant women diagnosed with overt hypothyroidism, 2,023 singleton pregnant women diagnosed with subclinical hypothyroidism, and 2,043 pregnant women with singleton pregnancies without thyroid-related complications or comorbidities, all of whom underwent NBS for amino acids and acylcarnitines. The participant inclusion criteria were as follows: (I) singleton pregnant women; (II) NBS for amino acids and acylcarnitines; and (III) delivery in Fujian Provincial Maternity and Child Hospital. The exclusion criteria were as follows: (I) multiple pregnancies; (II) stillbirth, neonatal death, fetal congenital abnormalities, and genetic metabolic diseases; (III) liver, heart, or renal disease; (IV) other perinatal complications, such as intrahepatic cholestasis during pregnancy and gestational diabetes mellitus; and (V) no NBS for amino acids and acylcarnitines.

Data were collected from the Fujian Maternity and Child Health Hospital between January 2016 and February 2024. **Figure 1** illustrates the workflow of the study design. This study was conducted in accordance with the guidelines of the Declaration of Helsinki and approved by the Ethics Review Committee of Fujian Provincial Maternity and Child Hospital (approval no.2024KY298), Fuzhou, China.

**Figure 1:**
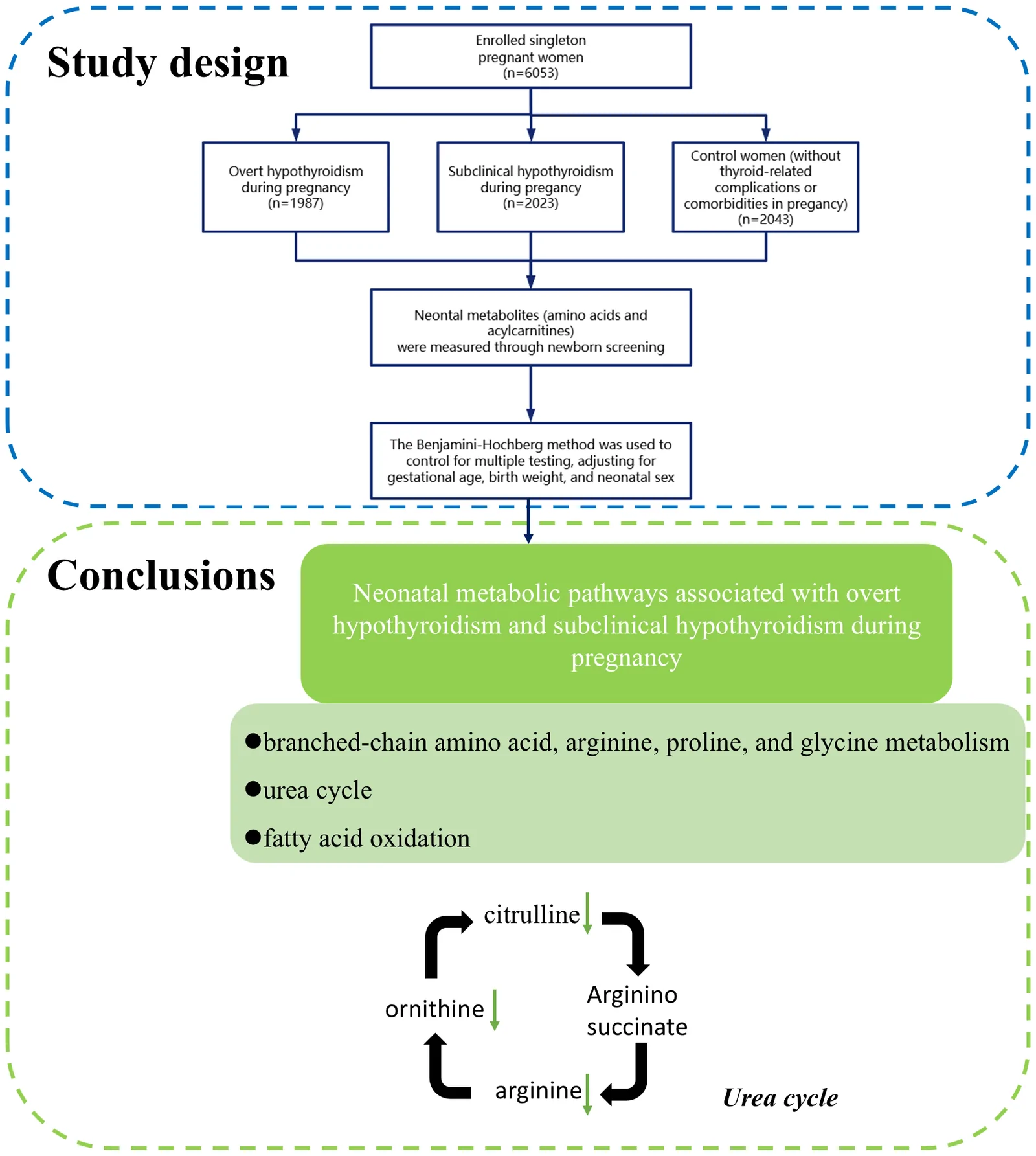
The workflow of the study design and neonatal metabolic pathways associated with overt hypothyroidism and subclinical hypothyroidism during pregnancy.

All participants diagnosed with overt or subclinical hypothyroidism during pregnancy immediately initiated standardized levothyroxine (L-T4) replacement therapy in accordance with the *Guideline on Diagnosis and Management of Thyroid Diseases during Pregnancy and Postpartum (2nd edition)* (2). The initial dosage was individualized according to the severity of thyroid dysfunction. Serum TSH and FT4 levels were regularly monitored during pregnancy, and the L-T4 dose was dynamically adjusted to maintain TSH within the pregnancy-specific reference range. Importantly, TSH levels were normalized to the gestational-specific reference range before delivery in all participants of the overt and subclinical hypothyroidism groups.

### 2.2 Thyroid function diagnostic criteria and clinical data collection

#### 2.2.1 Diagnostic criteria

Overt hypothyroidism during pregnancy: During pregnancy, the serum level of thyroid-stimulating hormone (TSH) exceeds the upper limit of the pregnancy-specific reference range, and the serum level of free tetraiodothyronine (FT4) is below the lower limit of the pregnancy-specific reference range (2). Subclinical hypothyroidism during pregnancy: During pregnancy, the serum TSH level exceeds the upper limit of the pregnancy-specific reference range, while the FT4 level remains within the pregnancy-specific reference range (2). Control group: Throughout pregnancy, thyroid function indicators (TSH and FT4) are within the pregnancy-specific reference ranges; there is no history of thyroid disease or family history of thyroid disease, and no other chronic diseases that may affect thyroid function.

#### 2.2.2 Clinical data collection

Clinical data of all subjects were collected, including general clinical information such as maternal age, gestational weeks, gestational age at delivery, neonatal birth weight, gender, and Apgar score; meanwhile, data related to neonatal heel blood screening were collected. All data were extracted from the hospital’s electronic medical record system by professionally trained medical staff and verified by two independent personnel to ensure data accuracy.

### 2.3 NBS and biochemical analysis

The newborn screening (NBS) procedure was performed in accordance with our previously described protocols (28, 29). Briefly, blood spot samples were collected from the heel of newborns, air-dried at room temperature (25°C), and transported to the NBS center in Fujian province using a cold-chain transportation system. The concentrations of amino acids and acylcarnitines in the dried blood spot samples were quantified using a tandem mass spectrometry (MS/MS) system (ACQUITY TQD, Waters, USA) with a NeoBase™ MS/MS Kit (PerkinElmer, Turku, Finland). Parameters of the liquid tandem mass spectrometer are shown in **Supplementary Table S1**. The amino acid profile included alanine, arginine, citrulline, glycine, leucine+isoleucine+hydroxyproline (Leu+Ile+Pro-OH), methionine, ornithine, phenylalanine, proline, valine, and tyrosine. The acylcarnitine profile included free carnitine (C0), acetylcarnitine (C2), propionylcarnitine (C3), malonylcarnitine + 3-hydroxybutyrylcarnitine (C3DC + C4OH), butyrylcarnitine + isobutyrylcarnitine (C4), methylmalonyl + 3-hydroxy-isovalerylcarnitine (C4DC + C5OH), isovalerylcarnitine + methylbutyrylcarnitine (C5), tiglylcarnitine (C5:1), glutarylcarnitine + 3-hydroxyhexanoylcarnitine (C5DC + C6OH), hexanoylcarnitine (C6), methylglutarylcarnitine (C6DC), octanoylcarnitine (C8), octenoylcarnitine (C8:1), decanoylcarnitine (C10), decenoylcarnitine (C10:1), decadienoylcarnitine (C10:2), dodecanoylcarnitine (C12), dodecenoylcarnitine (C12:1), tetradecanoylcarnitine (C14), tetradecenoylcarnitine (C14:1), tetradecadienoylcarnitine (C14:2), 3-hydroxy-tetradecanoylcarnitine (C14OH), palmitoylcarnitine (C16), palmitoleylcarnitine (C16:1), 3-hydroxy-hexadecanoylcarnitine (C16OH), 3-hydroxy-hexadecenoylcarnitine (C16:1OH), stearoylcarnitine (C18), oleoylcarnitine (C18:1), 3-hydroxy-octadecenoylcarnitine (C18:1OH), linoleoylcarnitine (C18:2), and 3-hydroxy-octadecanoylcarnitine (C18OH).

TSH concentrations in dried blood spots were determined by time-resolved fluorescence immunoassay (TRFIA) using a fully automated PerkinElmer GSP analyzer (Model 2021-0010) with matched commercial reagents. Internal quality control was performed in each batch with dual-level control materials in accordance with Westgard multi-rules. Local NBS reference ranges for all measured amino acids, acylcarnitines, and metabolite ratios are provided in **Supplementary Table S2**.

### 2.4 Statistical analyses

Statistical analyses were conducted with R software (version 4.5.2; https://www.r-project.org/) and IBM SPSS Statistics for Windows (version 25.0; IBM Corp., Armonk, N.Y., USA). Partial least squares discriminant analysis (PLS-DA) was conducted via the ropls package to characterize the overall distribution pattern of the data. To evaluate model overfitting and classification reliability, a permutation test was performed to calculate R²Y and Q² metrics for PLS-DA model validation. Permutation-based multivariate analysis of variance (PERMANOVA) was applied to assess the impacts of grouping, gestational age, birth weight, infant gender and age at heel prick on metabolite distribution. Volcano plots and heatmaps for differential metabolites were generated with the ggplot2 package and ComplexHeatmap package, respectively. Categorical data were expressed as frequency (n) and percentage (%), and chi-square test or Fisher’s exact test was used for intergroup comparisons of these data. For non-normally distributed continuous variables, median and interquartile range were adopted for description, and Kruskal–Wallis test was performed for multiple group comparisons. Dunn’s test with Bonferroni correction was utilized for pairwise *post-hoc* comparisons to identify the specific differences between groups. A P < 0.05 was regarded as statistically significant. Additionally, the Benjamini–Hochberg method was used for multiple testing correction, with the false discovery rate threshold set at 0.05.

## 3 Results

### 3.1 Basic clinical characteristics of the case-control study

A total of 6,053 women with singleton pregnancies and their newborns were enrolled in this study, comprising 1,987 women with overt hypothyroidism during pregnancy, 2,023 women with subclinical hypothyroidism during pregnancy, and 2,043 control women without any thyroid-related complications or comorbidities during pregnancy. The gestational age at delivery, birth weight, infant gender and age at heel prick are presented in **Table 1**.

**Table 1:** Comparisons of neonatal basic clinical data among mothers with overt hypothyroidism during pregnancy, subclinical hypothyroidism during pregnancy, and those without any thyroid-related complications or comorbidities during pregnancy.

Variable	Overt hypothyroidism during pregnancy group (N = 1987) Median or n (%)	Subclinical hypothyroidism during pregnancy group (N = 2023) Median or n (%)	Control group (N = 2043) Median or n (%)	Kruskal-Wallis H or χ^2^-Value	*P*
Gestational age at delivery (week)	39.3 (38.6,40.0)	39.4 (38.6,40.1)	39.7 (39.0,40.3)	109.709	**<0.001**
Birth weight (g)	3235 (2997.5,3490)	3255 (3010,3520)	3285 (3070,3530)	18.640	**<0.001**
Gender (male)	1052 (52.9%)	1076 (53.2%)	1102 (53.9%)	0.438	0.803
Age at heel prick (day)	3.29 (3.02,3.89)	3.33 (3.02,3.93)	3.45 (3.05,4.46)	46.956	**<0.001**

Table comparing baseline clinical data of neonates born to three maternal groups (overt hypothyroidism during pregnancy, subclinical hypothyroidism during pregnancy, control), presenting gestational age at delivery, birth weight and age at heel prick (both expressed as median and interquartile range), as well as the proportion of male neonates (n, %), along with Kruskal-Wallis H/χ² values and P values for intergroup statistical differences.Bold values indicate statistically significant differences (*P* < 0.05).

No significant differences in infant gender were observed among the three groups (P>0.05). However, significant differences in gestational age at delivery birth weight, and age at heel prick were observed among the three groups (P<0.001). Newborns born to mothers with overt hypothyroidism and subclinical hypothyroidism during pregnancy exhibited lower gestational ages, lowerbirth weights and an earlier age at heel prick than those born to mothers without any thyroid-related complications or comorbidities during pregnancy. Furthermore, we found that the birth weight in the overt hypothyroidism during pregnancy group was lower than that in the subclinical hypothyroidism during pregnancy group (P = 0.003). Additionally, no significant differences in gestational age or age at heel prick were found between the overt hypothyroidism during pregnancy group and the subclinical hypothyroidism during pregnancy group (P>0.05). The effects of group, gestational age, birth weight, infant gender and age at heel prick on metabolite distribution were evaluated using PERMANOVA. Significant inter-group differences were detected, as were significant effects for all other tested factors (**Table 2**).

**Table 2:** The results of PERMANOVA analysis among the overt hypothyroidism during pregnancy, subclinical hypothyroidism during pregnancy, and control groups.

Variable	Df	SumOfSqs	R2	F	Pr(>F)
Group	2	0.4216130	0.006271486	20.186759	**0.001**
Birth weight	1	0.1608123	0.002392080	15.399333	**0.001**
Gestational age at delivery	1	1.5158819	0.022548715	145.160327	**0.001**
Gender	1	0.3740817	0.005564458	35.821932	**0.001**
Age at heel prick	1	1.2523120	0.018628117	119.920959	**0.001**
Residual	6046	63.1372389	0.939165209		
Total	6052	67.2269781	1.000000000		

Table presenting PERMANOVA analysis results for neonates born to three maternal groups (overt hypothyroidism during pregnancy, subclinical hypothyroidism during pregnancy, control), including degrees of freedom (Df), sum of squares (SumOfSqs), R-squared (R2), F statistic and P value (Pr(>F)) for group, birth weight, gestational age at delivery, gender, age at heel prick, residual and total effects.Bold values indicate statistically significant differences (*P* < 0.05).

### 3.2 Comparisons of amino acid levels in the peripheral blood of newborns born to mothers with overt hypothyroidism during pregnancy, those born to mothers with subclinical hypothyroidism during pregnancy, and those born to control mothers

All samples from the three groups were analyzed by partial least squares discriminant analysis (PLS-DA). No clear separation among the three groups was observed in the PLS-DA plot. Model metrics including R2X, R2Y, Q2, and results from 100-iteration permutation testing are summarized in **Supplementary Table S3** and **Supplementary Figure S1**. Although the permutation test was statistically significant (pQ2 = 0.01), the low Q2 value (0.0493) suggested limited overall discriminatory power for group separation in this PLS-DA model. The top 15 variable importance in projection scores of the metabolites are presented in **Figure 2**. We further evaluated the impact of hypothyroidism during pregnancy (including overt and subclinical) on neonatal amino acid metabolism, the levels of 11 specific amino acids, as well as the ratios of tyrosine/phenylalanine and ornithine/citrulline. Except for phenylalanine, tyrosine, and tyrosine/phenylalanine ratio, significant differences were observed in the levels of the nine remaining amino acids, as well as the ratio of ornithine/citrulline among infants born to mothers with overt hypothyroidism during pregnancy, those born to mothers with subclinical hypothyroidism during pregnancy, and those born to control mothers (P<0.05) (**Supplementary Table S4**; **Figure 3A**). Pairwise comparisons among the overt hypothyroidism during pregnancy, subclinical hypothyroidism during pregnancy, and control groups (**Figure 4**) revealed that newborns born to mothers with overt hypothyroidism and subclinical hypothyroidism during pregnancy exhibited lower levels of alanine, Leu+Ile+Pro-OH, ornithine, proline, and valine, as well as the ratio of ornithine/citrulline, than those born to mothers in the control group, whereas the overall levels of arginine and glycine in these two groups were higher than those in the control group.

**Figure 2:**
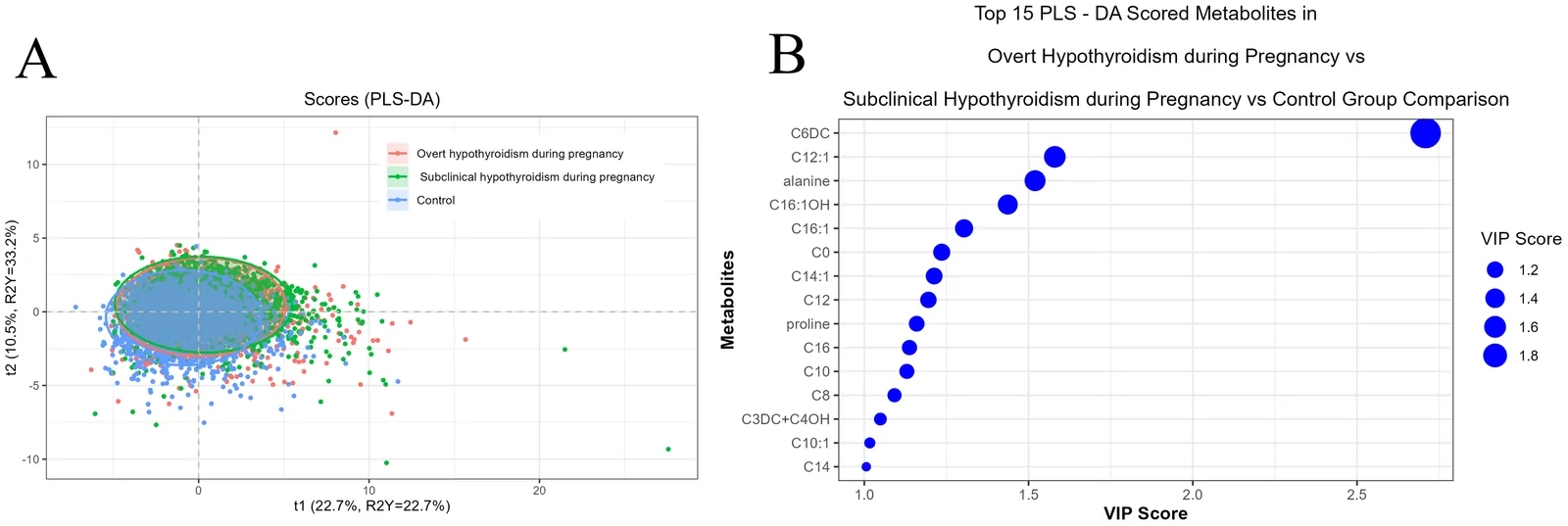
The PLS-DA analysis among the overt hypothyroidism during pregnancy, subclinical hypothyroidism during pregnancy, and control groups. **(A)** Overt hypothyroidism during pregnancy vs. Subclinical hypothyroidism during pregnancy vs. Control pregnancy. **(B)** The top 15 VIP scores of the metabolites in overt hypothyroidism during pregnancy vs. subclinical hypothyroidism during pregnancy, vs. control group comparison. VIP, variable importance in projection; PLS-DA, partial least squares discriminant analysis; C6DC, methylglutarylcarnitine; C12:1, dodecenoylcarnitine; C16:1OH, 3-hydroxypalmitoleylcarnitine; C16:1, palmitoleylcarnitine; C0, Carnitine; C14:1, tetradecenoylcarnitine; C12, dodecanoylcarnitine; C16, palmitoylcarnitine; C10, decanoylcarnitine; C8, octanoylcarnitine; C3DC+C4OH, malonylcarnitine + 3-Hydroxybutyrylcarnitine; C10:1, 3-Decenoylcarnitine; C14, tetradecanoylcarnitine.

**Figure 3:**
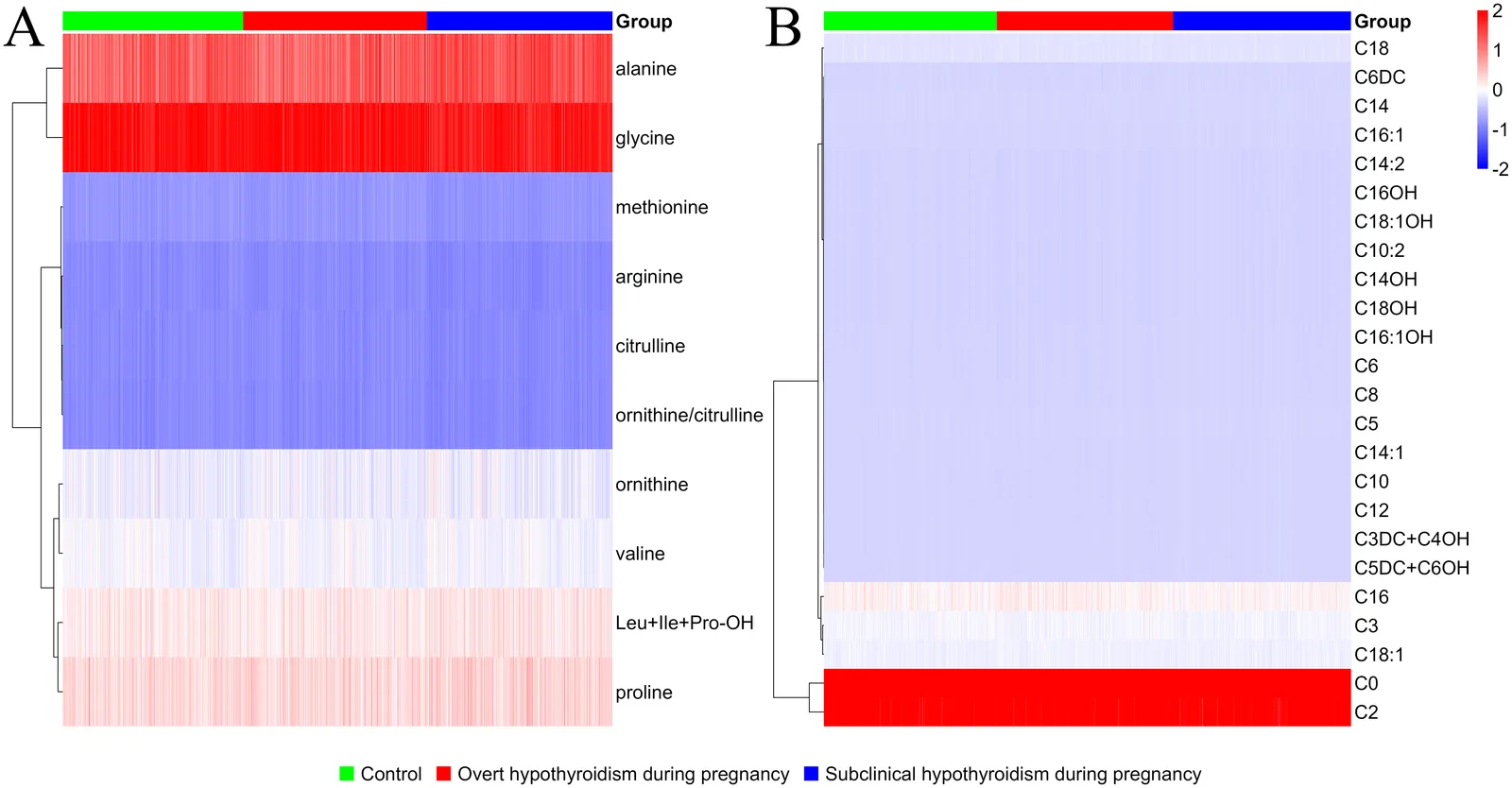
Heatmaps based on metabolite groups. **(A)** Amino acids; **(B)** Carnitines. Leu+Ile+Pro-OH, leucine+isoleucine+hydroxyproline; C0, free carnitine; C2, acetylcarnitine; C3, propionylcarnitine; C3DC + C4OH, malonylcarnitine + 3-hydroxybutyrylcarnitine; C5, isovalerylcarnitine + methylbutyrylcarnitine; C5DC + C6OH, glutarylcarnitine + 3-hydroxyhexanoylcarnitine; C6, hexanoylcarnitine; C6DC, methylglutarylcarnitine; C8, octanoylcarnitine; C10, decanoylcarnitine; C10:2, decadienoylcarnitine; C12, dodecanoylcarnitine; C14, tetradecanoylcarnitine; C14:1, tetradecenoylcarnitine; C14:2, tetradecadienoylcarnitine; C14OH, 3-hydroxy-tetradecanoylcarnitine; C16, palmitoylcarnitine; C16:1, palmitoleylcarnitine; C16:1OH, 3-hydroxy-hexadecenoylcarnitine; C16OH, 3-hydroxy-hexadecanoylcarnitine; C18, stearoylcarnitine; C18:1, oleoylcarnitine; C18:1OH, 3-hydroxy-octadecenoylcarnitine; C18OH, 3-hydroxy-octadecanoylcarnitine.

**Figure 4:**
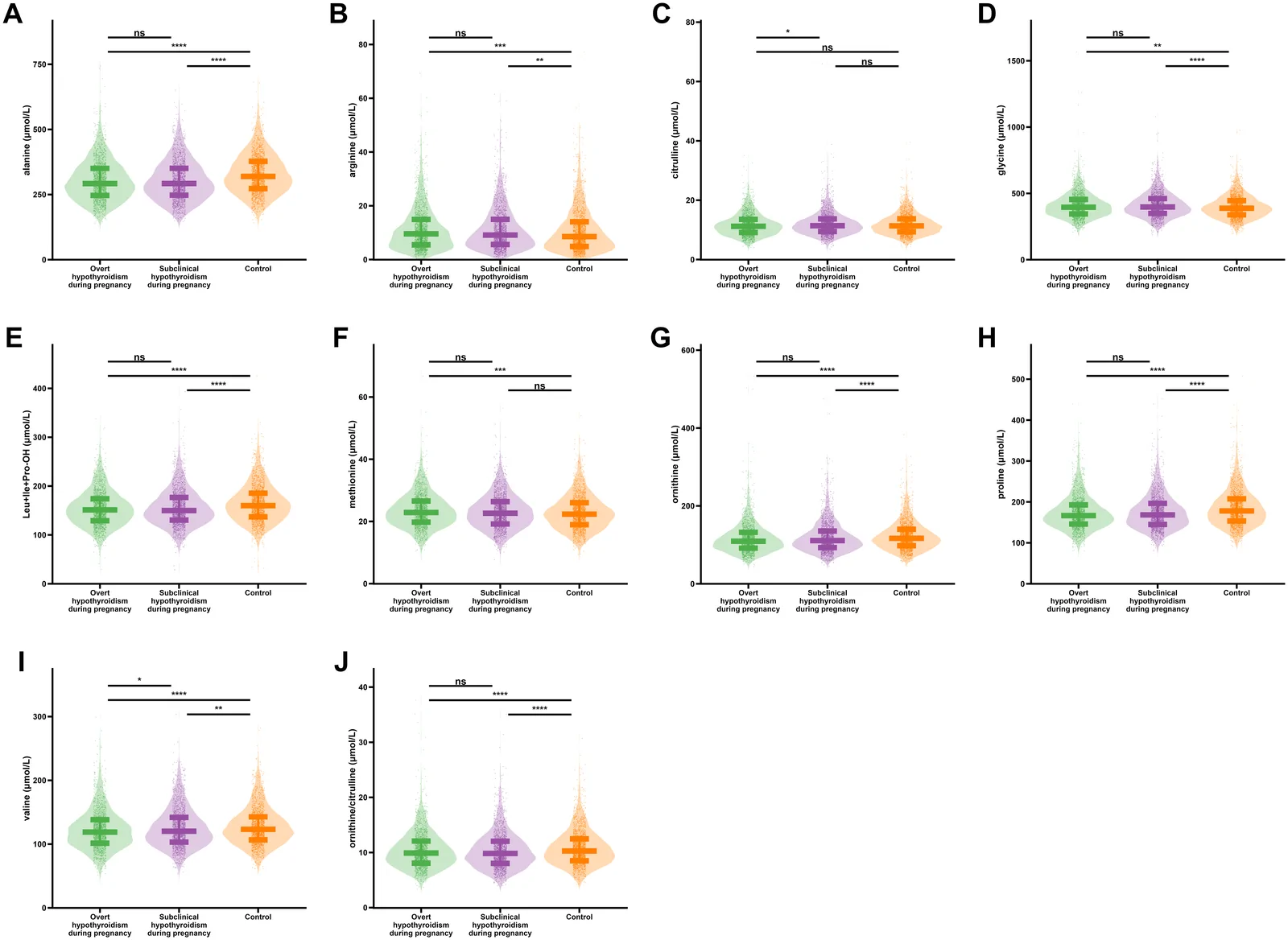
Pairwise comparisons of the levels of amino acids among the overt hypothyroidism during pregnancy, subclinical hypothyroidism during pregnancy, and control groups. **(A)** Alanine; **(B)** Arginine; **(C)** citrulline; **(D)** Glycine; **(E)** Leu+Ile+Pro-OH; **(F)** Methionine; **(G)** Ornithine; **(H)** Proline; **(I)** Valine; **(J)** Ornithine/citrulline ratio. Data are presented as median with interquartile range. *p < 0.05, **p < 0.01, ***p < 0.001, ****p < 0.0001. Leu+Ile+Pro-OH, leucine+isoleucine+hydroxyproline.

We performed sex-stratified analyses among the overt hypothyroidism during pregnancy, subclinical hypothyroidism during pregnancy, and control groups to explore sex-specific metabolite differences. Regardless of whether the infants were male or female, differences could be observed among the three groups in the overall alanine, arginine, glycine, Leu+Ile+Pro-OH, ornithine, proline and valine levels, as well as in the ornithine/citrulline ratios (**Supplementary Table S5**). We also performed the amino acids and birth weight correlation analyses. The results indicated that birth weight was positively correlated with Leu+Ile+Pro-OH, ornithine, proline, valine, as well as the ratio of ornithine/citrulline in the overt hypothyroidism during pregnancy group (P<0.05) (**Figure 5A**). In the subclinical hypothyroidism during pregnancy group, birth weight was positively correlated with Leu+Ile+Pro-OH, valine, and the ratio of ornithine/citrulline, but negatively correlated with citrulline, glycine, and tyrosine (P<0.05) (**Figure 5B**). In the control group, only Leu+Ile+Pro-OH, valine, and the ratio of ornithine/citrulline were positively correlated with birth weight (P<0.05) (**Figure 5C**). Meanwhile, the scatter plot of the statistically significant correlations between certain amino acids and birth weight in the three groups was presented in **Figure 5D**.

**Figure 5:**
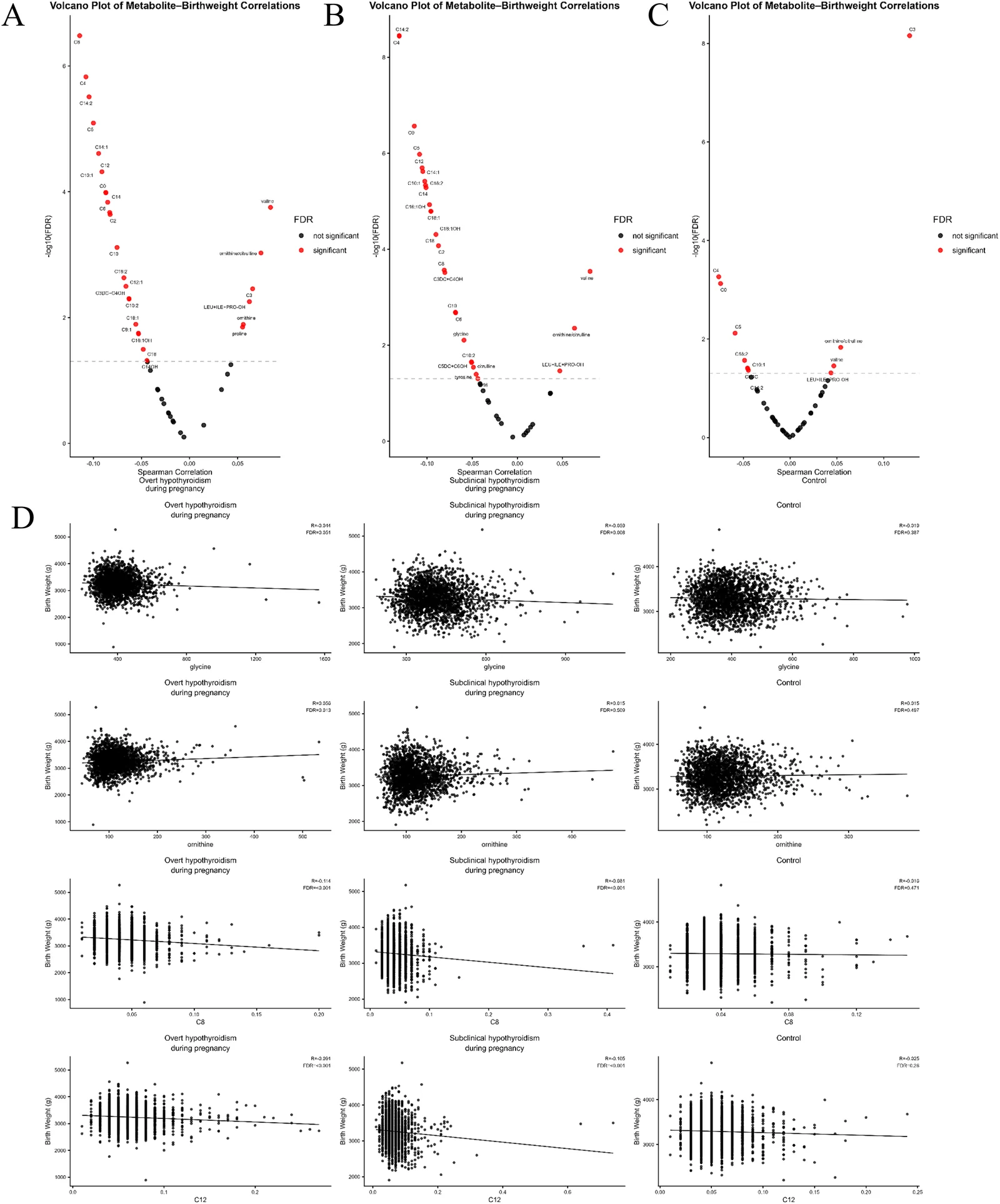
Correlation plot of metabolites with newborn birth weight among the overt hypothyroidism during pregnancy, subclinical hypothyroidism during pregnancy, and control groups. **(A)** Correlation plot of metabolites with newborn birth weight in the overt hypothyroidism during pregnancy group. **(B)** Correlation plot of metabolites with newborn birth weight in the subclinical hypothyroidism during pregnancy group. **(C)** Correlation plot of metabolites with newborn birth weight in the control group. **(D)** The scatter plot of top 4 pairwise correlations between metabolites and birth weight. C8, octanoylcarnitine; C12, dodecanoylcarnitine.

Gestational age and birth weight are considered to significantly influence amino acid metabolism in newborns (30–32). Thus, a linear regression analysis, adjusting for gestational age, birth weight, neonatal gender, and age at heel prick, revealed significant associations between the hypothyroidism during pregnancy group (including overt and subclinical) and amino acid levels (**Table 3**). After adjusting for multiple comparisons using the Benjamini–Hochberg method, alanine, Leu+Ile+Pro-OH, proline levels, and the ratio of ornithine/citrulline were significantly lower in the overt hypothyroidism during pregnancy, and subclinical hypothyroidism during pregnancy groups than in the control group. Conversely, arginine, glycine and methionine levels were significantly elevated in infants born to mothers in the two groups. Additionally, ornithine and valine levels were significantly lower in the overt hypothyroidism during pregnancy group than in the control group; while citrulline level was significantly higher in the subclinical hypothyroidism during pregnancy group than in the control group.

**Table 3:** Associations of overt and subclinical hypothyroidism during pregnancy with neonatal peripheral blood amino acid levels and selected ratios (with the control group as reference).

Amino acid	Overt hypothyroidism during pregnancy group (N = 1987)			Subclinical hypothyroidism during pregnancy group (N = 2023)		
	Std. β	β(95%CI)	FDR*	Std. β	β(95%CI)	FDR*
alanine	-0.112	-18.643(-23.292 to -13.994)	**<0.001**	-0.128	-21.169(-25.773 to -16.565)	**<0.001**
arginine	0.050	0.847(0.363 to 1.332)	**0.001**	0.041	0.697(0.217 to 1.177)	**0.007**
citrulline	–	0.003(-0.228 to 0.233)	0.982	0.036	0.291(0.063 to 0.520)	**0.018**
glycine	0.040	7.791(2.255 to 13.328)	**0.008**	0.064	12.328(6.843 to 17.812)	**<0.001**
Leu+Ile+Pro-OH	-0.090	-7.215(-9.481 to -4.948)	**<0.001**	-0.087	-6.929(-9.174 to -4.684)	**<0.001**
methionine	0.070	0.817(0.480 to 1.155)	**<0.001**	0.035	0.404(0.070 to 0.738)	**0.024**
ornithine	-0.050	-4.165(-6.535 to -1.795)	**0.001**	–	-2.464(-4.811 to -0.117)	0.053
phenylalanine	–	-0.039(-0.686 to 0.608)	0.973	–	0.509(-0.132 to 1.150)	0.154
proline	-0.098	-8.945(-11.567 to -6.323)	**<0.001**	-0.088	-7.999(-10.596 to -5.402)	**<0.001**
tyrosine	–	-0.272(-2.569 to 2.025)	0.921	–	0.618(-1.657 to 2.893)	0.670
valine	-0.046	-3.001(-4.836 to -1.166)	**0.002**	–	-0.867(-2.685 to 0.950)	0.405
tyrosine/phenylalanine ratio	–	-0.001(-0.046 to 0.044)	0.982	–	-0.004(-0.049 to 0.040)	0.884
ornithine/citrulline ratio	-0.048	-0.338(-0.543 to -0.133)	**0.002**	-0.059	-0.413(-0.615 to -0.210)	**<0.001**

FDR, false discovery rate; Leu+Ile+Pro-OH, leucine+isoleucine+hydroxyproline.

The models were adjusted for gestational age at delivery, birth weight, neonatal gender and age at heel prick. *FDR was calculated to account for multiple comparisons using the Benjamini-Hochberg method.

Table presenting regression analyses of associations between overt and subclinical hypothyroidism during pregnancy and neonatal peripheral blood amino acid levels, and selected amino acid ratios (with the control group as reference); shows standardized β (Std. β; reported only for statistically-significant comparisons), β coefficients (95% confidence interval, 95%CI) and false discovery rate (FDR) for the two hypothyroidism groups separately, with all analytical models adjusted for gestational age at delivery, birth weight, neonatal gender and age at heel prick.Bold values indicate statistically significant differences (*P* < 0.05).

### 3.3 Comparisons of acylcarnitine levels in the peripheral blood of newborns born to mothers with overt hypothyroidism during pregnancy, those born to mothers with subclinical hypothyroidism during pregnancy, and those born to control mothers

To assess the effects of hypothyroidism during pregnancy (including overt and subclinical) on metabolic disturbances in acylcarnitine profiles, the levels of 31 acylcarnitine levels were measured and compared between newborns of mothers with hypothyroidism during pregnancy (including overt and subclinical) and those of mothers in the control group. Except for C4, C4DC + C5OH, C5:1, C8:1, C10:1, C12:1, and C18:2, significant differences were observed in the levels of the remaining acylcarnitines among the three groups (P < 0.05) (**Supplementary Table S4**; **Figure 3B**). Pairwise comparisons of the 24 acylcarnitines among the overt hypothyroidism during pregnancy, the subclinical hypothyroidism during pregnancy and the control groups are illustrated in **Figure 6**. Newborns from the hypothyroidism during pregnancy (including overt and subclinical) groups exhibited higher levels of C2, C3, C3DC + C4OH, C5DC + C6OH, C6, C8, C10, C10:2, C12, C14, C14:1, C14:2, C14OH, C16, C16:1, C16OH, C18, C18:1, C18:1OH, and C18OH than those from the control group (P<0.05). In addition, C0, C5, C6DC, and C16:1OH levels were significantly lower in newborns from the hypothyroidism during pregnancy (including overt and subclinical) groups than in those from the control group. In the comparison of two hypothyroidism during pregnancy (including overt and subclinical) groups, the levels of C16, C16:1, C16OH and C18:1OH in newborns from the overt hypothyroidism during pregnancy group were all lower than those in newborns from the subclinical hypothyroidism during pregnancy group, whereas the C6DC level was significantly elevated in newborns from the overt hypothyroidism during pregnancy group.

**Figure 6:**
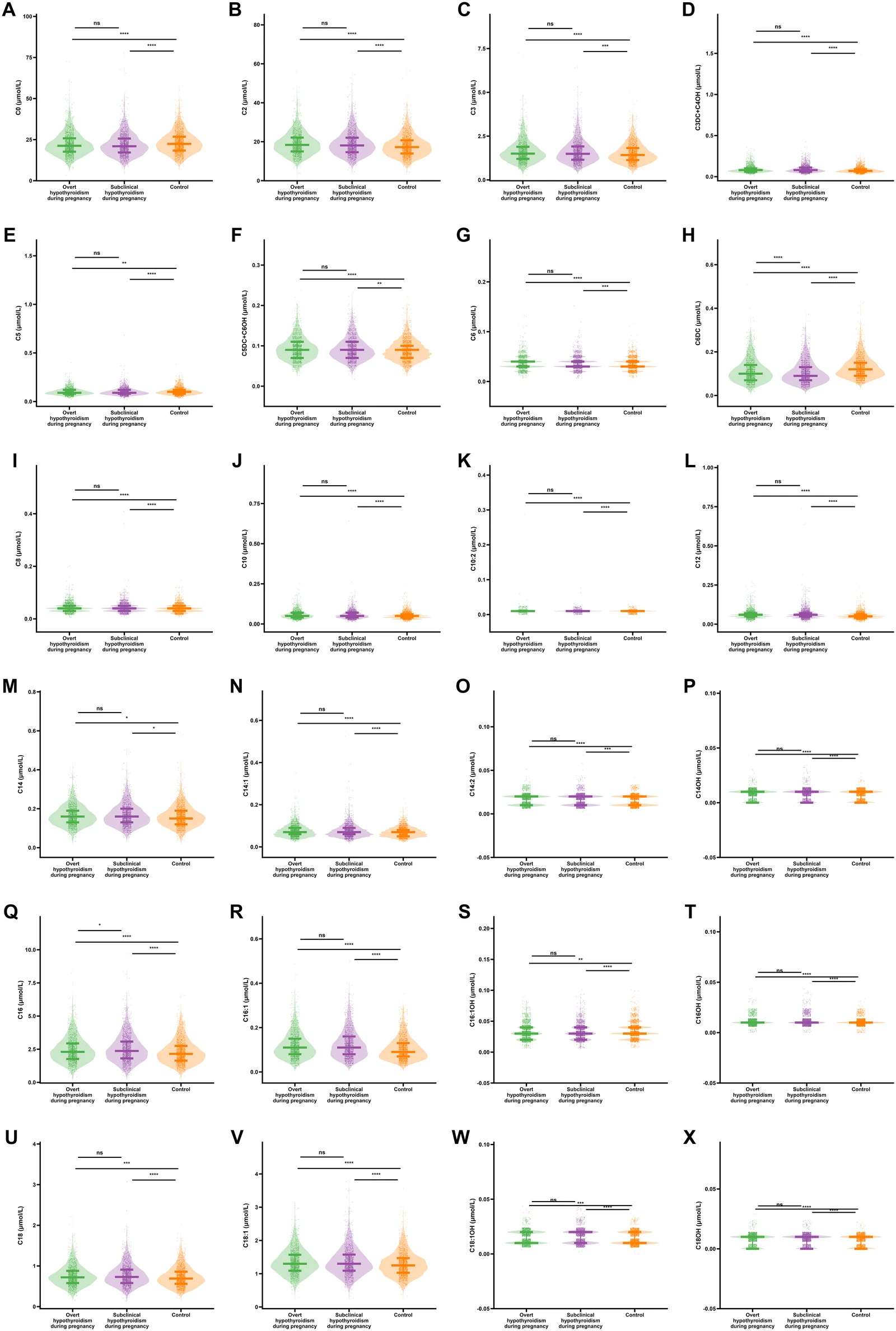
Pairwise comparisons of the levels of acylcarnitines among the overt hypothyroidism during pregnancy, subclinical hypothyroidism during pregnancy, and control groups. **(A)** C0; **(B)** C2; **(C)** C3; **(D)** C3DC+C4OH; **(E)** C5; **(F)** C5DC+C6OH; **(G)** C6; **(H)** C6DC; **(I)** C8; **(J)** C10; **(K)** C10:2; **(L)** C12; **(M)** C14; **(N)** C14:1; **(O)** C14:2; **(P)** C14OH; **(Q)** C16; **(R)** C16:1; **(S)** C16:1OH; **(T)** C16OH; **(U)** C18; **(V)** C18:1; **(W)** C18:1OH; **(X)** C18OH. Data are presented as median with interquartile range. *p < 0.05, **p < 0.01, ***p < 0.001, ****p < 0.0001. C0, free carnitine; C2, acetylcarnitine; C3, propionylcarnitine; C3DC + C4OH, malonylcarnitine + 3-hydroxybutyrylcarnitine; C5, isovalerylcarnitine + methylbutyrylcarnitine; C5DC + C6OH, glutarylcarnitine + 3-hydroxyhexanoylcarnitine; C6, hexanoylcarnitine; C6DC, methylglutarylcarnitine; C8, octanoylcarnitine; C10, decanoylcarnitine; C10:2, decadienoylcarnitine; C12, dodecanoylcarnitine; C14, tetradecanoylcarnitine; C14:1, tetradecenoylcarnitine; C14:2, tetradecadienoylcarnitine; C14OH, 3-hydroxy-tetradecanoylcarnitine; C16, palmitoylcarnitine; C16:1, palmitoleylcarnitine; C16:1OH, 3-hydroxy-hexadecenoylcarnitine; C16OH, 3-hydroxy-hexadecanoylcarnitine; C18, stearoylcarnitine; C18:1, oleoylcarnitine; C18:1OH, 3-hydroxy-octadecenoylcarnitine; C18OH, 3-hydroxy-octadecanoylcarnitine.

In addition, **Supplementary Table S5** presents the acylcarnitine level-related differences between the three groups for male or female infants. We also performed the acylcarnitine and birth weight correlation analyses. The results showed that birth weight was significantly correlated with C0, C2, C3DC+C4OH, C4, C5, C5DC+C6OH, C6, C8, C10, C10:1, C10:2, C12, C14, C14:1, C14:2, C16, C16:1OH, C18, C18:1, C18:1OH, and C18:2 in the overt hypothyroidism during pregnancy group (P < 0.05) (**Figure 5A**). Birth weight was significantly correlated with C0, C2, C3DC+C4OH, C4, C5, C6, C8, C8:1, C10, C10:1, C10:2, C12, C12:1, C14, C14:1, C14:2,C14OH, C16:1OH, C18, C18:1, C18:2, and C3 in the subclinical hypothyroidism during pregnancy group (**Figure 5B**), while only C3, C0, C4, C5, C6DC, C10:1,C14:2, and C18:2 were significantly correlated with birth weight in the control group (**Figure 5C**). Meanwhile, the scatter plot of the statistically significant correlations between certain acylcarnitines and birth weight in the three groups was also presented in **Figure 5D**. After adjusting for gestational age and birth weight, as well as neonatal gender and age at heel prick, the levels of C2, C3, C3DC+C4OH, C5DC+C6OH, C6, C8, C10, C10:2, C12, C14:1, C14:2, C14OH, C16, C16:1, C16OH, C18, C18:1, C18:1OH, and C18OH were significantly higher in newborns from the hypothyroidism during pregnancy (including overt and subclinical) groups than in those from the control group, while the levels of C0, C5, C6DC and C16:1OH were significantly lower in newborns from the two groups than in those from the control group (**Supplementary Table S6**).

Furthermore, we analyzed pairwise correlations across metabolites in the overt hypothyroidism during pregnancy group, the subclinical hypothyroidism during pregnancy group and the control group, respectively. The results were presented in **Figures 7A–C**. **Figure 7D** shows the top 10 differential pairwise correlations across metabolites among the overt hypothyroidism during pregnancy group, the subclinical hypothyroidism during pregnancy group and the control group, as well as between the overt hypothyroidism during pregnancy group and the subclinical hypothyroidism during pregnancy group. Meanwhile, scatter plots of pairwise correlations across certain different metabolites among the three groups are shown in **Figure 7E**.

**Figure 7:**
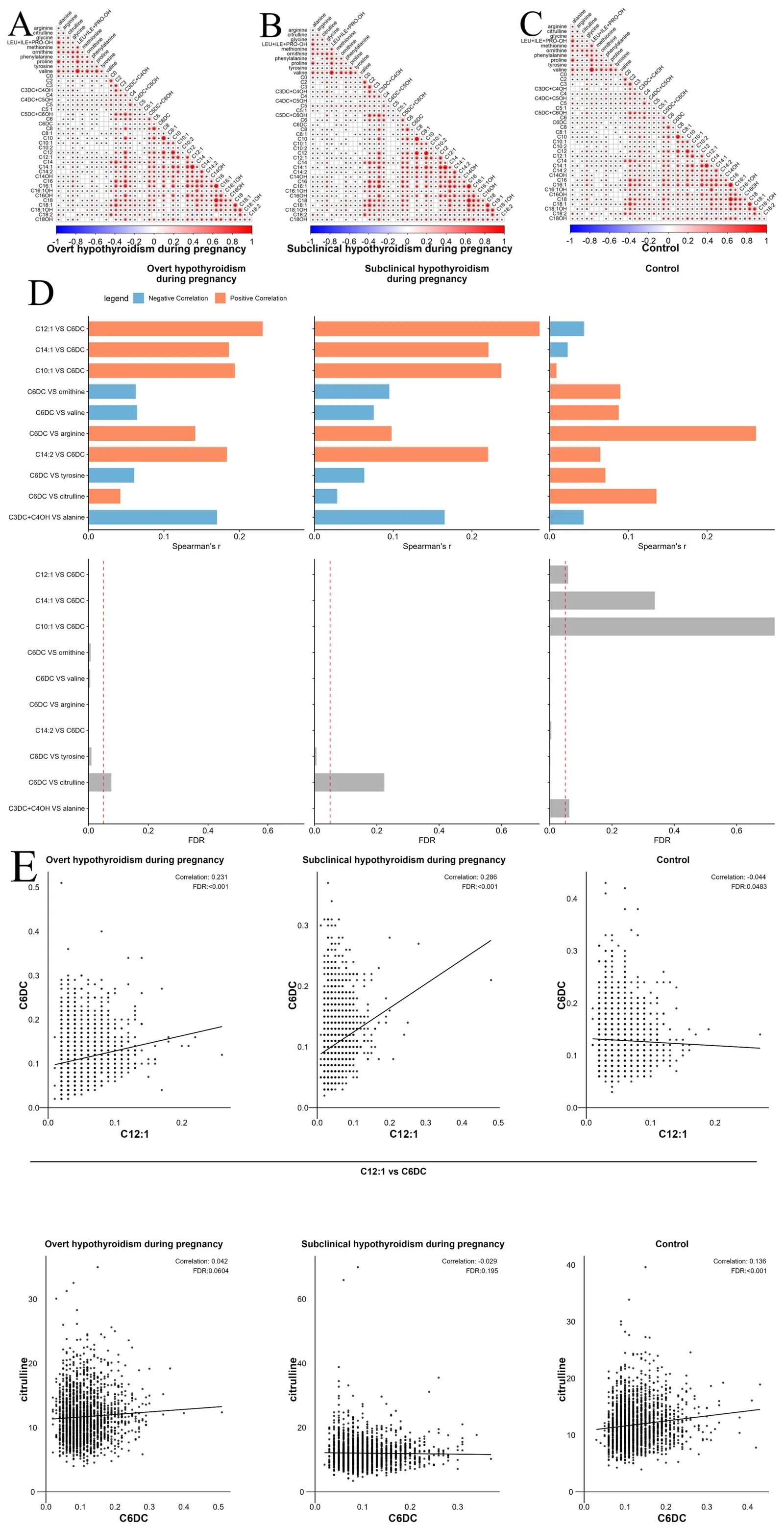
Pairwise correlations across metabolites in the overt hypothyroidism during pregnancy, subclinical hypothyroidism during pregnancy, and control groups. Correlations specific to each group are highlighted by a rectangle. **(A)** Pairwise correlations across metabolites in the overt hypothyroidism during pregnancy group. **(B)** Pairwise correlations across metabolites in the subclinical hypothyroidism during pregnancy group. **(C)** Pairwise correlations across metabolites in the control group. **(D)** The top 10 different pairwise correlations across metabolites among the overt hypothyroidism during pregnancy, subclinical hypothyroidism during pregnancy, and control groups. **(E)** The scatter plot of different pairwise correlations among the overt hypothyroidism during pregnancy, subclinical hypothyroidism during pregnancy, and control groups. Leu+Ile+Pro-OH, leucine+isoleucine+hydroxyproline; C0, free carnitine; C2, acetylcarnitine; C3, propionylcarnitine; C3DC + C4OH, malonylcarnitine + 3-hydroxybutyrylcarnitine; C4, butyrylcarnitine + isobutyrylcarnitine; C4DC + C5OH, methylmalonyl + 3-hydroxy-isovalerylcarnitine; C5, isovalerylcarnitine + methylbutyrylcarnitine; C5:1, tiglylcarnitine; C5DC + C6OH, glutarylcarnitine + 3-hydroxyhexanoylcarnitine; C6, hexanoylcarnitine; C6DC, methylglutarylcarnitine; C8, octanoylcarnitine; C8:1, octenoylcarnitine; C10, decanoylcarnitine; C10:1, decenoylcarnitine; C10:2, decadienoylcarnitine; C12, dodecanoylcarnitine; C12:1, dodecenoylcarnitine; C14, tetradecanoylcarnitine; C14:1, tetradecenoylcarnitine; C14:2, tetradecadienoylcarnitine; C14OH, 3-hydroxy-tetradecanoylcarnitine; C16, palmitoylcarnitine; C16:1, palmitoleylcarnitine; C16:1OH, 3-hydroxy-hexadecenoylcarnitine; C16OH, 3-hydroxy-hexadecanoylcarnitine; C18, stearoylcarnitine; C18:1, oleoylcarnitine; C18:1OH, 3-hydroxy-octadecenoylcarnitine; C18:2, linoleoylcarnitine; C18OH, 3-hydroxy-octadecanoylcarnitine.

### 3.4 Comparisons of thyroid-stimulating hormone level in the peripheral blood of newborns born to mothers with overt hypothyroidism during pregnancy, those born to mothers with subclinical hypothyroidism during pregnancy, and those born to control mothers

A total of 1,987 newborns were enrolled in the overt hypothyroidism during pregnancy group in this study, among whom TSH values were missing in 3 cases (missing rate: 0.15%). Considering that the missing samples were concentrated in the same group with an extremely low missing rate, median imputation was performed using the complete TSH values within the overt hypothyroidism during pregnancy group: the median of all complete TSH records in the overt hypothyroidism during pregnancy group was first calculated (2.54 mIU/L), and then this value was filled in the 3 missing positions to maximize the consistency with the actual TSH levels of the missing samples and reduce data deviation.

A comparison of TSH levels in newborns from the overt hypothyroidism during pregnancy group, the subclinical hypothyroidism during pregnancy group and the control group revealed that TSH levels in newborns from the overt hypothyroidism during pregnancy group and the subclinical hypothyroidism during pregnancy group were both higher than those in newborns from the control group (P<0.001) (**Table 4**). In contrast, comparison of the two hypothyroidism during pregnancy (including overt and subclinical) groups revealed no statistically significant difference in TSH levels of newborns between the two groups (P = 0.484).

**Table 4:** Comparison of TSH levels among newborns in the overt hypothyroidism during pregnancy, subclinical hypothyroidism during pregnancy, and control groups.

Variable	Overt hypothyroidism during pregnancy group (N = 1987) Median (P25, P75)	Subclinical hypothyroidism during pregnancy group (N = 2023) Median (P25, P75)	Control group (N = 2043) Median (P25, P75)	Kruskal-Wallis H	*P*	FDR	ε²
TSH	2.54 (1.50, 4.03)	2.56 (1.50, 4.20)	2.00 (1.15, 3.30)	136.212	**<0.001**	**<0.001**	**0.023**

FDR, false discovery rate.

Table presenting comparisons of TSH levels among newborns in the maternal overt hypothyroidism during pregnancy, subclinical hypothyroidism during pregnancy and control groups; shows median values (25th, 75th percentiles) for each group, with Kruskal-Wallis H statistic, P value, false discovery rate (FDR), and epsilon-squared (ϵ²; reported only for statistically-significant comparisons) effect-size values provided for intergroup differences.Bold values indicate statistically significant differences (*P* < 0.05).

## 4 Discussion

Expanded NBS, which incorporates MS/MS technology, offers a fundamental metabolic profile for newborns and serves as a panel of biomarkers indicative of fetal growth and overall health. Numerous fetal and maternal factors may influence the metabolites detected through NBS. However, beyond gestational age and birth weight, the understanding of the underlying etiologies affecting these measurements at birth remains limited. This study utilized a targeted metabolomics platform to analyze the blood metabolites of newborns born to mothers with hypothyroidism during pregnancy (including overt and subclinical hypothyroidism) using MS/MS analysis. Our findings revealed that several metabolites differed significantly between newborns of mothers with hypothyroidism during pregnancy (including overt and subclinical hypothyroidism) and those of mothers without any thyroid-related complications or comorbidities during pregnancy.

As a glucogenic amino acid, alanine supports glucose synthesis, counteracts hypoglycemia, and aids in maintaining energy balance within the organism. Fetal alanine deficiency may compromise energy generation, contributing to intrauterine growth restriction and increasing the risk of hypoglycemia after birth (33, 34). In the present study, blood alanine concentrations were significantly lower in the hypothyroidism during pregnancy (including overt and subclinical hypothyroidism) group than in the control group. In addition, stratified analysis by sex revealed that this difference existed in both male and female neonates, suggesting a universal association of hypothyroidism during pregnancy (including overt and subclinical hypothyroidism) with altered fetal alanine metabolism.

The branched-chain amino acids (BCAAs)—leucine, valine, and isoleucine—are three essential proteinogenic amino acids characterized by their aliphatic side-chain structure. As essential amino acids, BCAAs have strong insulinogenic and anabolic effects, and have been reported to mediate lean body mass growth via the mammalian target of rapamycin (mTOR) signaling pathway in prior studies (35, 36). These described biological functions are consistent with their potential role in fetal metabolic regulation. Leucine, as a core branched-chain amino acid and essential nutrient, has been shown in previous work to act as a key signaling molecule that activates the mammalian target of rapamycin complex 1 (mTORC1) pathway to promote skeletal muscle protein synthesis. It also participates in energy metabolism via branched-chain amino acid aminotransferase (BCAT)/branched-chain α-ketoacid dehydrogenase (BCKDH)-mediated catabolism (37). In the present study, neonates in the hypothyroidism during pregnancy group (encompassing overt and subclinical hypothyroidism) exhibited significantly decreased levels of Leu+Ile+Pro-OH and valine. This finding may reflect a specific association between maternal hypothyroidism and altered fetal BCAAs metabolism, and BCAAs deficiency could represent a potential correlate linked to fetal growth restriction.

Arginine, a conditionally essential amino acid during mammalian pregnancy, promotes embryonic and fetal survival and growth by synthesizing nitric oxide and polyamines, activating the mTOR pathway, and improving utero-placental blood flow and nutrient transport (38). For mothers with overt hypothyroidism or subclinical hypothyroidism during pregnancy, elevated TSH may induce a compensatory increase in placental type 3 deiodinase (D3) activity, which catalyzes triiodothyronine (T3) inactivation and further reduces fetal T3 supply (39, 40). As thyroid hormones have been reported to regulate placental trophoblast transport function (41), arginine is known to be actively transported from mother to fetus across the placenta (42), and feto-placental amino-acid uptake and release are well-described physiological processes in human gestation (43). Drawing upon these published observations, one may speculate that fetal T3 insufficiency could weaken the inhibitory effect of T3 on placental arginine transport, potentially contributing to fetal arginine accumulation. Nevertheless, this mechanistic chain remains to be experimentally verified. Our study found that neonates born to the hypothyroidism during pregnancy groups (encompassing overt and subclinical hypothyroidism) had significantly higher arginine levels than those in the control group. This elevation of neonatal arginine levels may reflect perturbed placental arginine metabolism and transport linked to maternal thyroid hormone insufficiency and could serve as a potential metabolic indicator of fetal adaptive responses to intrauterine thyroid hormone deficiency.

Ornithine and citrulline are key intermediates in the urea cycle. Within mitochondria, ornithine is catalyzed by ornithine transcarbamylase (OTC) to form citrulline, which is then transported to the cytoplasm via ornithine transporter 1 (ORNT1) for subsequent metabolic processes, and both compounds are involved in ammonia metabolism and detoxification *in vivo*. Aberrant levels of these metabolites can lead to ammonia accumulation and cause potential damage to the fetal nervous system (44). Our study revealed that the neonatal ornithine level in both the overt hypothyroidism during pregnancy group and the subclinical hypothyroidism during pregnancy group was significantly lower than that in the control group, whereas the citrulline level in the subclinical hypothyroidism during pregnancy group was significantly higher than that in the overt hypothyroidism during pregnancy group. Moreover, the ornithine/citrulline ratio was significantly decreased in both groups. These findings suggest that maternal hypothyroidism may be specifically associated with altered fetal-urea-cycle-related metabolite profiles.

Glycine exerts anti-oxidative stress and immune-regulatory effects and is involved in the synthesis of essential biomolecules including glutathione and purines in the fetus, and its altered levels may compromise cellular protective mechanisms and thereby elevate the risk of oxidative damage (45). Methionine, by contrast, acts as a key substrate for methylation modifications and participates in the regulation of gene expression as well as fetal growth and development; its deficiency or excess can disrupt normal physiological processes, leading to impairments in DNA methylation patterns and aberrant gene expression (46). Consistent with the metabolic regulatory role of thyroid hormones, our study revealed significantly higher levels of glycine and methionine in neonates born to mothers with overt or subclinical hypothyroidism compared with the control group, a finding that suggests maternal hypothyroidism may be associated with shifts in the fetal internal environment, which could potentially involve oxidative-stress-related and methylation-related metabolic perturbations. The elevated levels of glycine and methionine may represent a defensive response of the fetus to counteract oxidative damage, while their persistent altered elevation may exert adverse effects on gene expression and cellular functions.

Proline is involved in fetal collagen synthesis and tissue repair and is also closely associated with energy metabolism. A decrease in its levels may impair fetal tissue development and growth rate, serving as a potential risk factor for low birth weight and possibly leading to hypoplasia of tissues such as the skin and skeleton (47). In the present study, neonatal proline levels were significantly lower in both the overt hypothyroidism during pregnancy group and the subclinical hypothyroidism during pregnancy group than in the control group. Furthermore, proline levels were positively correlated with birth weight in the overt hypothyroidism during pregnancy group, suggesting an association between altered proline metabolism and fetal growth restriction. Based on these observations, one may speculate that maternal hypothyroidism could be linked to insufficient proline supply via altered proline synthesis or placental transport, potentially compromising tissue development and fetal growth, although this proposed mechanistic pathway awaits further experimental validation.

Changes in acylcarnitine levels in neonates may be attributed to the synergistic effects of multiple factors from the maternal, fetal and placental compartments. Thyroid hormones serve as key regulators of fatty acid metabolism. Specifically, they can activate key enzymes including carnitine palmitoyltransferases (CPT1/2) and mitochondrial trifunctional protein (MTP); meanwhile, they relieve the inhibitory effect of acetyl-CoA carboxylase (ACC) through the adenosine 5’-monophosphate-activated protein kinase (AMPK) signaling pathway. These regulatory effects collectively promote the acylcarnitine-mediated import and oxidation of mitochondrial fatty acids, thereby maintaining the balance of acylcarnitine metabolism. In contrast, thyroid hormone deficiency impairs the activity of the aforementioned enzymes and reduces the efficiency of relevant signaling pathways, which disrupts this metabolic balance and ultimately results in acylcarnitine metabolic disorders (48). Our study found that except for a total of 7 acylcarnitines including C4, C4DC+C5OH, C5:1, C8:1, C10:1, C12:1 and C18:2, significant differences were observed in the remaining 24 acylcarnitines among the overt hypothyroidism during pregnancy group, the subclinical hypothyroidism during pregnancy group, and the control group.

TSH is the core hormone regulating thyroid function. An elevation in serum TSH levels usually indicates insufficient secretion of peripheral thyroid hormones, and the pituitary gland compensatorily increases TSH secretion through a negative feedback mechanism. The normal development of fetal thyroid function relies on the supply of maternal thyroid hormones. When maternal hypothyroidism occurs, the transfer of thyroid hormones to the fetus via the placenta decreases. The reduction in fetal thyroid hormone levels stimulates the fetal pituitary gland to secrete more TSH through negative-feedback regulation of the hypothalamic-pituitary-thyroid axis, aiming to compensatorily maintain thyroid function (49). However, the fetal thyroid gland is immature with limited compensatory capacity and cannot fully counteract reduced metabolic enzyme activity. Combined with our present observations, these endocrine disturbances may partly explain the alterations in amino-acid and acylcarnitine profiles seen in neonates born to hypothyroid mothers.

This study highlights potential clinical relevance of maternal hypothyroidism during pregnancy (including overt and subclinical forms) by demonstrating observed associations between this maternal condition and neonatal amino acid and acylcarnitine metabolic abnormalities, as well as elevated TSH levels. It also identifies potential key abnormal indicators, which may offer candidate markers for clinical reference. Furthermore, the study offers data support and practical implications for enhancing prenatal thyroid function screening and implementing standardized treatment, which may help optimize the placental metabolic microenvironment and lower the risk of fetal metabolic disorders.

Although this study presents valuable findings, several limitations should be acknowledged. As a retrospective study, selection bias may be present. In addition, patients with gestational diabetes and hypertensive disorders of pregnancy were excluded from our cohort; accordingly, our findings cannot be generalized to pregnant women with overt or subclinical hypothyroidism who have these gestational complications. While all participants with overt or subclinical hypothyroidism in our cohort received standardized levothyroxine replacement therapy and achieved gestation-specific TSH targets before delivery, data regarding gestational age at hypothyroidism diagnosis, timing of levothyroxine initiation, and pretreatment baseline thyroid hormone levels were unavailable. We therefore cannot evaluate fetal thyroid-hormone exposure during the interval between disease onset and biochemical normalization, and the observed associations cannot be interpreted independently of the potential effects of treatment. Moreover, several important maternal clinical covariates, including maternal BMI, thyroid peroxidase antibody (TPOAb) status, parity, and iodine status, were not routinely recorded within the NBS database and could not be adjusted for in our analyses, raising the risk of residual confounding. The study sample was derived from a single region, which may limit the generalizability of the results. Furthermore, long-term neonatal health data were not collected, so the long-term impact of these observed metabolic alterations cannot be clarified. Further prospective studies with complete records of diagnosis, treatment-initiation timing, and comprehensive maternal clinical indicators are needed to address these limitations.

In conclusion, this study revealed a significant association between maternal clinical and subclinical hypothyroidism during pregnancy and neonatal amino acid and acylcarnitine levels, which were mainly involved in branched-chain amino acid, arginine, proline, and glycine metabolism, the urea cycle pathway, a feto-placental and fatty acid oxidation pathway (**Figure 1**).

## Data Availability

The original contributions presented in the study are included in the article/**Supplementary Material**. Further inquiries can be directed to the corresponding authors.

## Supplementary material

Supplementary Figure 1 Permutation test for the three-group PLS-DA model based on 100 permutations. Alt text: Permutation test plot for the three-group PLS-DA model (100 permutations), showing distributions of permuted R2Y and Q2Y metrics.
