# Supplementary material for: Overt and subclinical hypothyroidism during pregnancy: effects on neonatal amino acids, acylcarnitines, and TSH levels
Source: Front Endocrinol (Lausanne). 2026 Sep 25;17:1872893. doi: 10.3389/fendo.2026.1872893 (PMC13649493; doi:10.3389/fendo.2026.1872893)
Supplement: Supplementary file 1 [file Table1.docx]

**Table S1. The parameters of the liquid tandem mass spectrometer.**

| Compound Name | Parent (m/z) | Daughter (m/z) | Dwell (s) | Cone (v) | Collision (v) |
| --- | --- | --- | --- | --- | --- |
| glycine | 76 | 30 | 0.050 | 20 | 7 |
| glycine IS | 78 | 32 | 0.050 | 20 | 7 |
| alanine | 90.1 | 44 | 0.050 | 20 | 8 |
| alanine IS | 94.1 | 48 | 0.050 | 20 | 8 |
| proline | 116.1 | 70.1 | 0.025 | 12 | 14 |
| proline IS | 121.1 | 74.1 | 0.025 | 12 | 14 |
| valine | 118.1 | 72.1 | 0.025 | 22 | 12 |
| valine IS | 126.1 | 80.1 | 0.025 | 22 | 12 |
| Leu+Ile+Pro-OH | 132.1 | 86.1 | 0.025 | 21 | 10 |
| Leu+Ile+Pro-OH IS | 135.1 | 89.1 | 0.025 | 21 | 10 |
| ornithine | 133.2 | 70.1 | 0.025 | 18 | 16 |
| ornithine IS | 139.2 | 76.1 | 0.025 | 18 | 16 |
| methionine | 150.1 | 104.1 | 0.025 | 19 | 10 |
| methionine IS | 153.1 | 107.1 | 0.025 | 19 | 10 |
| phenylalanine | 166.1 | 120.1 | 0.025 | 22 | 13 |
| phenylalanine IS | 172.1 | 126.1 | 0.025 | 22 | 13 |
| citrulline | 176.1 | 113.1 | 0.025 | 19 | 16 |
| citrulline IS | 178.1 | 115.1 | 0.025 | 19 | 16 |
| arginine | 175.2 | 70.1 | 0.025 | 28 | 20 |
| arginine IS | 180.2 | 75.1 | 0.025 | 28 | 20 |
| tyrosine | 182.1 | 136.1 | 0.025 | 21 | 13 |
| tyrosine IS | 188.1 | 142.1 | 0.025 | 21 | 13 |
| C0 | 162.2 | 103 | 0.025 | 32 | 15 |
| C0 IS | 171.2 | 103 | 0.025 | 32 | 15 |
| C2 | 204.2 | 85 | 0.025 | 29 | 18 |
| C2 IS | 207.2 | 85 | 0.025 | 29 | 18 |
| C3 | 218.2 | 85 | 0.025 | 29 | 19 |
| C3 IS | 221.2 | 85 | 0.025 | 29 | 19 |
| C4 | 232.2 | 85 | 0.025 | 30 | 19 |
| C4 IS | 235.2 | 85 | 0.025 | 30 | 19 |
| C5:1 | 244.2 | 85 | 0.025 | 30 | 21 |
| C5 | 246.2 | 85 | 0.025 | 30 | 21 |
| C3DC+C4OH | 248.2 | 85 | 0.025 | 30 | 19 |
| C5 IS | 255.2 | 85 | 0.025 | 30 | 21 |
| C6 | 260.2 | 85 | 0.025 | 31 | 21 |
| C4DC+C5OH | 262.2 | 85 | 0.025 | 30 | 21 |
| C6 IS | 263.2 | 85 | 0.025 | 31 | 21 |
| C5DC+C6OH | 276.2 | 85 | 0.05 | 31 | 22 |
| C5DC/C6OH IS | 282.2 | 85 | 0.05 | 31 | 22 |
| C8:1 | 286.3 | 85 | 0.025 | 33 | 22 |
| C8 | 288.3 | 85 | 0.025 | 33 | 22 |
| C6DC | 290.2 | 85 | 0.050 | 31 | 22 |
| C8 IS | 291.3 | 85 | 0.025 | 33 | 22 |
| C10:2 | 312.3 | 85 | 0.025 | 37 | 24 |
| C10:1 | 314.3 | 85 | 0.025 | 37 | 24 |
| C10 | 316.3 | 85 | 0.025 | 37 | 24 |
| C10 IS | 319.3 | 85 | 0.025 | 37 | 24 |
| C12:1 | 342.3 | 85 | 0.025 | 40 | 26 |
| C12 | 344.3 | 85 | 0.025 | 40 | 26 |
| C12 IS | 347.3 | 85 | 0.025 | 40 | 26 |
| C14:2 | 368.3 | 85 | 0.025 | 43 | 27 |
| C14:1 | 370.3 | 85 | 0.025 | 43 | 27 |
| C14 | 372.3 | 85 | 0.025 | 43 | 27 |
| C14 IS | 375.3 | 85 | 0.025 | 43 | 27 |
| C14OH | 388.3 | 85 | 0.025 | 43 | 27 |
| C16:1 | 398.4 | 85 | 0.025 | 45 | 28 |
| C16 | 400.4 | 85 | 0.025 | 45 | 28 |
| C16 IS | 403.4 | 85 | 0.025 | 45 | 28 |
| C16:1OH | 414.4 | 85 | 0.025 | 45 | 28 |
| C16OH | 416.4 | 85 | 0.025 | 45 | 28 |
| C18:2 | 424.4 | 85 | 0.025 | 48 | 29 |
| C18:1 | 426.4 | 85 | 0.025 | 48 | 29 |
| C18 | 428.4 | 85 | 0.025 | 48 | 29 |
| C18 IS | 431.4 | 85 | 0.025 | 48 | 29 |
| MS/MS condition | | 3 |  |  |  |
| Capillary(KV) | | 3 |  |  |  |
| Extractor(V) | | 0.1 |  |  |  |
| RF Lens(V) | | 120 |  |  |  |
| Source Temp(℃) | | 350 |  |  |  |
| Desolvation Temp(℃) | | 650 |  |  |  |
| Desolvation Gas Flow(L/hr) | | 20 |  |  |  |
| Cone Gas Flow(L/hr) | | 3 |  |  |  |
| LC Conditions | |  |  |  |  |
| Liquid Phase Gradient | | Mobile Phase 100% (Pump A or Pump B); 0.00-0.18 min,0.20mL/min;0.18-1.00min,0.02mL/min;1.00-1.5min,0.70mL/min. | | | |
| Washes | | 50/50 methanol/Water | | | |
| Inject volume | | 20μL |  |  |  |

Abbreviation: Leu+Ile+Pro-OH: leucine+isoleucine+hydroxyproline, C0: free carnitine, C2: acetylcarnitine, C3: propionylcarnitine, C3DC + C4OH: malonylcarnitine + 3-hydroxybutyrylcarnitine, C4: butyrylcarnitine + isobutyrylcarnitine, C4DC + C5OH: methylmalonyl + 3-hydroxy-isovalerylcarnitine, C5: isovalerylcarnitine + methylbutyrylcarnitine, C5:1: tiglylcarnitine, C5DC + C6OH: glutarylcarnitine + 3-hydroxyhexanoylcarnitine, C6: hexanoylcarnitine, C6DC: methylglutarylcarnitine, C8: octanoylcarnitine, C8:1: octenoylcarnitine, C10: decanoylcarnitine, C10:1: decenoylcarnitine, C10:2: decadienoylcarnitine, C12: dodecanoylcarnitine, C12:1: dodecenoylcarnitine, C14: tetradecanoylcarnitine, C14:1: tetradecenoylcarnitine, C14:2: tetradecadienoylcarnitine, C14OH: 3-hydroxy-tetradecanoylcarnitine, C16: palmitoylcarnitine, C16:1: palmitoleylcarnitine, C16OH: 3-hydroxy-hexadecanoylcarnitine, C16:1OH: 3-hydroxy-hexadecenoylcarnitine, C18: stearoylcarnitine, C18:1: oleoylcarnitine, C18:1OH: 3-hydroxy-octadecenoylcarnitine, C18:2: linoleoylcarnitine, C18OH: 3-hydroxy-octadecanoylcarnitine.

Alt text: Comprehensive table listing liquid tandem mass spectrometer parameters for amino acids, acylcarnitines and their internal standards (IS), including parent/daughter m/z, dwell time, cone and collision voltage; also presents MS/MS and LC operating conditions such as capillary voltage, source temperature, mobile phase gradient and inject volume.
