# Supplementary material for: Overt and subclinical hypothyroidism during pregnancy: effects on neonatal amino acids, acylcarnitines, and TSH levels
Source: Front Endocrinol (Lausanne). 2026 Sep 25;17:1872893. doi: 10.3389/fendo.2026.1872893 (PMC13649493; doi:10.3389/fendo.2026.1872893)
Supplement: Supplementary file 2 [file Table2.docx]

**Table S2 Reference ranges of metabolites from the Fujian provincial newborn screening program.**

| Metabolite | Reference ranges (μmol/L) |
| --- | --- |
| alanine | 130-650 |
| arginine | 1-50 |
| citrulline | 6.1-30 |
| glycine | 180-1000 |
| Leu+Ile+Pro-OH | 65-300 |
| methionine | 8.5-45 |
| ornithine | 40-350 |
| phenylalanine | 25-115 |
| proline | 89-480 |
| tyrosine | 30-350 |
| valine | 60-250 |
| phenylalanine/tyrosine | 0.15-1.4 |
| ornithine/citrulline ratio | 2.5-22 |
| C0 | 8.8-50 |
| C2 | 4.5-51 |
| C3 | 0.3-4 |
| C3DC + C4OH | 0.02-0.35 |
| C4 | 0.08-0.5 |
| C4DC + C5OH | 0.07-0.39 |
| C5 | 0.03-0.35 |
| C5:1 | 0-0.02 |
| C5DC + C6OH | 0.03-0.2 |
| C6 | 0-0.08 |
| C6DC | 0.03-0.33 |
| C8 | 0.01-0.12 |
| C8:1 | 0.03-0.3 |
| C10 | 0.02-0.2 |
| C10:1 | 0-0.14 |
| C10:2 | 0-0.4 |
| C12 | 0.02-0.22 |
| C12:1 | 0.01-0.23 |
| C14 | 0.04-0.4 |
| C14:1 | 0.02-0.25 |
| C14:2 | 0-0.04 |
| C14OH | 0-0.04 |
| C16 | 0.5-6.5 |
| C16:1 | 0.02-0.48 |
| C16:1OH | 0-0.08 |
| C16OH | 0-0.04 |
| C18 | 0.2-2 |
| C18:1 | 0.4-3 |
| C18:1OH | 0-0.04 |
| C18:2 | 0.05-0.64 |
| C18OH | 0-0.02 |
