# Supplementary material for: Overt and subclinical hypothyroidism during pregnancy: effects on neonatal amino acids, acylcarnitines, and TSH levels
Source: Front Endocrinol (Lausanne). 2026 Sep 25;17:1872893. doi: 10.3389/fendo.2026.1872893 (PMC13649493; doi:10.3389/fendo.2026.1872893)
Supplement: Supplementary file 3 [file Table3.docx]

**Table S3 Model performance parameters of the partial least‑squares discriminant analysis (PLS‑DA) and permutation test results.**

| **Parameter** | **Value** |
| --- | --- |
| Number of components | 2 |
| R2X | 0.332 |
| R2Y | 0.0515 |
| Q2 | 0.0493 |
| Permutation times | 100 |
| pQ2 | 0.01 |

*Note:* R2X, cumulative explained variation of the X‑matrix; R2Y, cumulative explained variation of the Y‑matrix; Q2, predictive ability of the model obtained by cross‑validation; Permutation times, iteration number of permutation test; pQ2, p‑value derived from permutation validation.
