# Supplementary material for: Overt and subclinical hypothyroidism during pregnancy: effects on neonatal amino acids, acylcarnitines, and TSH levels
Source: Front Endocrinol (Lausanne). 2026 Sep 25;17:1872893. doi: 10.3389/fendo.2026.1872893 (PMC13649493; doi:10.3389/fendo.2026.1872893)
Supplement: Supplementary file 4 [file Table4.docx]

**Table S4 Comparisons of amino acid and acylcarnitine levels in peripheral blood of newborns born to mothers with hypothyroidism** **(including overt and subclinical) during pregnancy and** **those without any thyroid-related complications or comorbidities during pregnancy.**

| **Amino acid or Acylcarnitine** | **Overt hypothyroidism during pregnancy group (N=1987)**  **Median (P25, P75)** | **Subclinical hypothyroidism during pregnancy group**  **(N=2023)**  **Median (P25, P75)** | **Control group**  **(N=2043)**  **Median (P25, P75)** | **Kruskal-Wallis H** | ***P*** | **FDR** | **ε²** |
| --- | --- | --- | --- | --- | --- | --- | --- |
| alanine | 292.14(246.655,350.31) | 292.32(247.515,350.675) | 319.66(272.98,377.57) | 158.648 | **＜0.001** | **＜0.001** | **0.026** |
| arginine | 9.57(5.47,14.91) | 9.10(5.60,14.95) | 8.54(4.86,14.07) | 16.974 | **＜0.001** | **＜0.001** | **0.003** |
| citrulline | 11.20(9.115,13.52） | 11.41(9.435,13.71) | 11.35(9.36,13.72) | 6.817 | **0.033** | **0.043** | **0.001** |
| glycine | 395.43(344.92,453.94) | 397.43(348.71,460.88) | 388.22(338.42,445.01) | 22.596 | **＜0.001** | **＜0.001** | **0.004** |
| Leu+Ile+Pro-OH | 151.26(129.035,174.005) | 149.90(130.275,176.715) | 159.90(137.10,185.39) | 85.864 | **＜0.001** | **＜0.001** | **0.014** |
| methionine | 22.86(19.775,26.555) | 22.63(19.19,26.37) | 22.34(18.96,26.02) | 14.277 | **0.001** | **0.001** | **0.002** |
| ornithine | 109.18(91.475,132.11) | 110.95(92.945,135.485) | 116.73(97.82,140.02) | 53.354 | **＜0.001** | **＜0.001** | **0.009** |
| phenylalanine | 49.33(42.985,56.465) | 50.04(43.99,57.08) | 49.27(43.16,56.33) | 5.826 | 0.054 | 0.064 | - |
| proline | 166.58(145.945,192.78) | 168.33(144.86,196.47) | 178.13(153.41,207.71) | 93.695 | **＜0.001** | **＜0.001** | **0.015** |
| tyrosine | 92.44(74.21,115.32) | 91.78(74.16,117.50) | 90.71(72.70,114.89) | 3.255 | 0.196 | 0.213 | - |
| valine | 118.85(101.42,138.36) | 120.19(103.215,141.92) | 123.24(106.69,142.81) | 36.005 | **＜0.001** | **＜0.001** | **0.006** |
| tyrosine/  phenylalanine ratio | 1.8593(1.4861,2.3392) | 1.8439(1.4761,2.3289) | 1.84(1.47,2.31) | 1.085 | 0.581 | 0.581 | - |
| ornithine/  citrulline ratio | 9.9079(8.0712,12.0787) | 9.8313(8.0266,12.0421) | 10.28(8.50,12.48) | 31.693 | **＜0.001** | **＜0.001** | **0.005** |
| C0 | 21.26(17.65, 25.79) | 20.96(17.18, 25.67) | 22.39(18.37, 26.77) | 49.051 | **＜0.001** | **＜0.001** | **0.008** |
| C2 | 18.37(15.01, 22.13) | 18.09(14.63, 22.06) | 17.22(13.98, 20.74) | 51.626 | **＜0.001** | **＜0.001** | **0.009** |
| C3 | 1.50(1.20, 1.89) | 1.49(1.15, 1.91) | 1.42(1.13, 1.82) | 24.485 | **＜0.001** | **＜0.001** | **0.004** |
| C3DC + C4OH | 0.08(0.06, 0.10) | 0.08(0.06, 0.11) | 0.07(0.06, 0.09) | 98.627 | **＜0.001** | **＜0.001** | **0.016** |
| C4 | 0.19(0.16, 0.23) | 0.19(0.16, 0.23) | 0.19(0.16, 0.23) | 0.869 | 0.648 | 0.648 | - |
| C4DC + C5OH | 0.17(0.14, 0.20) | 0.17(0.14, 0.20) | 0.17(0.14, 0.20) | 2.592 | 0.274 | 0.293 | - |
| C5 | 0.09(0.08, 0.12) | 0.09(0.08, 0.12) | 0.10(0.08, 0.12) | 19.165 | **＜0.001** | **＜0.001** | **0.003** |
| C5:1 | 0.00(0.00,0.01) | 0.00(0.00,0.01) | 0.00(0.00,0.01) | 3.991 | 0.136 | 0.150 | - |
| C5DC + C6OH | 0.09(0.07, 0.11) | 0.09(0.07, 0.11) | 0.09(0.07, 0.10) | 21.459 | **＜0.001** | **＜0.001** | **0.004** |
| C6 | 0.04(0.03, 0.04) | 0.03(0.03, 0.04) | 0.03(0.03, 0.04) | 22.390 | **＜0.001** | **＜0.001** | **0.004** |
| C6DC | 0.10(0.07, 0.14) | 0.09(0.07, 0.13) | 0.12(0.09, 0.15) | 340.668 | **＜0.001** | **＜0.001** | **0.056** |
| C8 | 0.04(0.03, 0.05) | 0.04(0.03, 0.05) | 0.04(0.03, 0.05) | 47.085 | **＜0.001** | **＜0.001** | **0.008** |
| C8:1 | 0.10(0.08, 0.12) | 0.10(0.08, 0.12) | 0.10(0.08, 0.12) | 1.196 | 0.550 | 0.568 | - |
| C10 | 0.05(0.04, 0.07) | 0.05(0.04, 0.07) | 0.05(0.04, 0.06) | 54.707 | **＜0.001** | **＜0.001** | **0.009** |
| C10:1 | 0.06(0.05, 0.07) | 0.06(0.04, 0.07) | 0.06(0.04, 0.07) | 4.161 | 0.125 | 0.143 | - |
| C10:2 | 0.01(0.01, 0.01) | 0.01(0.01, 0.01) | 0.01(0.01, 0.01) | 57.883 | **＜0.001** | **＜0.001** | **0.010** |
| C12 | 0.06(0.05, 0.07) | 0.06(0.05, 0.07) | 0.05(0.04, 0.06) | 148.605 | **＜0.001** | **＜0.001** | **0.025** |
| C12:1 | 0.04(0.03, 0.07) | 0.04(0.03, 0.06) | 0.04(0.03, 0.06) | 4.956 | 0.084 | 0.104 | - |
| C14 | 0.16(0.13, 0.19) | 0.16(0.13, 0.20) | 0.15(0.12, 0.19) | 8.654 | **0.013** | **0.017** | **0.001** |
| C14:1 | 0.07(0.06, 0.09) | 0.07(0.06, 0.09) | 0.07(0.05, 0.08) | 32.038 | **＜0.001** | **＜0.001** | **0.005** |
| C14:2 | 0.02(0.01, 0.02) | 0.02(0.01, 0.02) | 0.02(0.01, 0.02) | 27.479 | **＜0.001** | **＜0.001** | **0.005** |
| C14OH | 0.01(0.00, 0.01) | 0.01(0.00, 0.01) | 0.01(0.00, 0.01) | 82.524 | **＜0.001** | **＜0.001** | **0.014** |
| C16 | 2.30(1.77, 2.94) | 2.37(1.81, 3.08) | 2.15(1.64, 2.75) | 70.183 | **＜0.001** | **＜0.001** | **0.012** |
| C16:1 | 0.11(0.08, 0.15) | 0.11(0.08, 0.16) | 0.09(0.07, 0.13) | 137.929 | **＜0.001** | **＜0.001** | **0.023** |
| C16:1OH | 0.03(0.02, 0.04) | 0.03(0.02, 0.04) | 0.03(0.03, 0.04) | 22.381 | **＜0.001** | **＜0.001** | **0.004** |
| C16OH | 0.01(0.01, 0.01) | 0.01(0.01, 0.01) | 0.01(0.01, 0.01) | 66.644 | **＜0.001** | **＜0.001** | **0.011** |
| C18 | 0.72(0.58, 0.88) | 0.73(0.58, 0.91) | 0.69(0.56, 0.86) | 32.659 | **＜0.001** | **＜0.001** | **0.005** |
| C18:1 | 1.30(1.09, 1.57) | 1.30(1.09, 1.58) | 1.25(1.03, 1.47) | 40.579 | **＜0.001** | **＜0.001** | **0.007** |
| C18:1OH | 0.01(0.01, 0.02) | 0.02(0.01, 0.02) | 0.01(0.01, 0.02) | 37.909 | **＜0.001** | **＜0.001** | **0.006** |
| C18:2 | 0.23(0.18, 0.30) | 0.23(0.18, 0.30) | 0.24(0.18, 0.30) | 4.684 | 0.096 | 0.115 | - |
| C18OH | 0.01(0.00, 0.01) | 0.01(0.00, 0.01) | 0.01(0.00, 0.01) | 54.481 | **＜0.001** | **＜0.001** | **0.009** |

Abbreviation: FDR, false discovery rate; Leu+Ile+Pro-OH, leucine+isoleucine+hydroxyproline; C0, free carnitine; C2, acetylcarnitine; C3, propionylcarnitine; C3DC + C4OH, malonylcarnitine + 3-hydroxybutyrylcarnitine; C4, butyrylcarnitine + isobutyrylcarnitine; C4DC + C5OH, methylmalonyl + 3-hydroxy-isovalerylcarnitine; C5, isovalerylcarnitine + methylbutyrylcarnitine; C5:1, tiglylcarnitine; C5DC + C6OH, glutarylcarnitine + 3-hydroxyhexanoylcarnitine; C6, hexanoylcarnitine; C6DC, methylglutarylcarnitine; C8, octanoylcarnitine; C8:1, octenoylcarnitine; C10, decanoylcarnitine; C10:1, decenoylcarnitine; C10:2, decadienoylcarnitine; C12, dodecanoylcarnitine; C12:1, dodecenoylcarnitine; C14, tetradecanoylcarnitine; C14:1, tetradecenoylcarnitine; C14:2, tetradecadienoylcarnitine; C14OH, 3-hydroxy-tetradecanoylcarnitine; C16, palmitoylcarnitine; C16:1, palmitoleylcarnitine; C16:1OH, 3-hydroxy-hexadecenoylcarnitine; C16OH, 3-hydroxy-hexadecanoylcarnitine; C18, stearoylcarnitine; C18:1, oleoylcarnitine; C18:1OH, 3-hydroxy-octadecenoylcarnitine; C18:2, linoleoylcarnitine; C18OH, 3-hydroxy-octadecanoylcarnitine.

Alt text: Table comparing peripheral blood amino acid, acylcarnitine levels, and selected amino acid ratios in neonates born to three maternal groups (overt hypothyroidism during pregnancy, subclinical hypothyroidism during pregnancy, control), with all indices expressed as median (25th, 75th percentile); presents Kruskal-Wallis H statistic, P value, false discovery rate (FDR), and epsilon‑squared (ε²**;** reported only for statistically‑significant comparisons) effect-size values for intergroup statistical differences.
