# Supplementary material for: Overt and subclinical hypothyroidism during pregnancy: effects on neonatal amino acids, acylcarnitines, and TSH levels
Source: Front Endocrinol (Lausanne). 2026 Sep 25;17:1872893. doi: 10.3389/fendo.2026.1872893 (PMC13649493; doi:10.3389/fendo.2026.1872893)
Supplement: Supplementary file 5 [file Table5.docx]

**Table S5** **The sex-stratified analyses of amino acid and acylcarnitine levels in peripheral blood of newborns** **among the overt hypothyroidism during pregnancy, subclinical hypothyroidism during pregnancy, and control groups.**

| **Amino acid or Acylcarnitine** | **Male** | | | | | | | **Female** | | | | | | |
| --- | --- | --- | --- | --- | --- | --- | --- | --- | --- | --- | --- | --- | --- | --- |
|  | **Overt hypothyroidism during pregnancy group (N=1987)**  **Median (P25, P75)** | **Subclinical hypothyroidism during pregnancy group**  **(N=2023)**  **Media (P25, P75)** | **Control group**  **(N=2043)**  **Median (P25, P75)** | **Kruskal-Wallis H** | ***P*** | **FDR** | **ε²** | **Overt hypothyroidism during pregnancy group (N=1987)**  **Median (P25, P75)** | **Subclinical hypothyroidism during pregnancy group**  **(N=2023)**  **Median (P25, P75)** | **Control group**  **(N=2043)**  **Median (P25, P75)** | **Kruskal-Wallis H** | ***P*** | **FDR** | **ε²** |
| alanine | 285.73(240.40, 338.49) | 286.33(243.30, 342.88) | 310.12(263.70, 365.61) | 74.267 | ＜0.001 | **＜0.001** | 0.023 | 302.05(253.44, 358.05) | 301.80(252.76, 360.18) | 330.94(283.89, 391.92) | 88.877 | ＜0.001 | **＜0.001** | **0.031** |
| arginine | 9.58(5.70, 14.75) | 9.77(5.73, 15.63) | 8.74(5.00, 14.70) | 9.303 | 0.010 | **0.012** | 0.003 | 9.53(5.29, 15.02) | 8.62(5.40, 14.03) | 8.25(4.73, 13.34) | 11.282 | 0.004 | **0.006** | **0.004** |
| citrulline | 10.89(8.89, 13.05) | 11.22(9.29, 13.46) | 11.24(9.15, 13.54) | 10.600 | 0.005 | **0.008** | 0.003 | 11.61(9.42, 14.04) | 11.73(9.63, 14.03) | 11.45(9.65, 14.02) | 0.140 | 0.933 | 0.933 | - |
| glycine | 384.60(336.52, 438.24) | 390.25(339.73, 453.88) | 381.36(329.33, 438.83) | 11.069 | 0.004 | **0.007** | 0.003 | 408.55(355.48, 466.00) | 409.68(356.87, 468.26) | 396.47(350.66, 453.71) | 12.955 | 0.002 | **0.003** | **0.005** |
| Leu+Ile+  Pro-OH | 148.39(126.94, 170.33) | 147.96(127.98, 173.18) | 157.39(135.43, 181.29) | 43.233 | ＜0.001 | **＜0.001** | 0.013 | 154.37(131.71, 178.58) | 152.50(132.54, 178.33) | 163.18(140.57, 189.19) | 44.077 | ＜0.001 | **＜0.001** | **0.016** |
| methionine | 22.19(19.03, 25.87) | 21.95(18.69, 25.93) | 21.66(18.07, 25.09) | 9.609 | 0.008 | **0.012** | 0.003 | 23.75(20.49, 27.31) | 23.40(20.04, 26.70) | 23.10(19.98, 26.80) | 6.094 | 0.048 | 0.069 | - |
| ornithine | 107.87(90.06, 130.99) | 109.77(91.77, 135.68) | 115.42(97.27, 139.38) | 32.355 | **＜0.001** | **＜0.001** | 0.010 | 110.50(93.02, 134.25) | 112.17(94.06, 135.42) | 117.58(98.49, 140.64) | 22.336 | ＜0.001 | **＜0.001** | **0.008** |
| phenylalanine | 49.56(42.88, 56.42) | 50.42(44.23, 56.74) | 48.85(42.59, 55.93) | 8.073 | 0.018 | **0.021** | 0.003 | 49.01(43.11, 56.53) | 49.56(43.70, 57.57) | 49.68(43.68, 56.83) | 2.048 | 0.359 | 0.424 | - |
| proline | 164.57(144.22, 190.16) | 167.65(143.27, 195.69) | 177.00(152.36, 204.67) | 53.063 | ＜0.001 | **＜0.001** | 0.016 | 168.34(148.06, 195.03) | 169.26(146.74, 197.40) | 180.66(154.22, 210.70) | 41.758 | ＜0.001 | **＜0.001** | **0.015** |
| tyrosine | 90.37(71.85, 113.13) | 91.17(73.61, 116.87) | 87.70(69.52, 112.28) | 7.494 | 0.024 | **0.026** | 0.002 | 93.62(76.21, 117.48) | 92.24(74.62, 118.20) | 93.72(75.86, 117.55) | 0.638 | 0.727 | 0.788 | - |
| valine | 115.05(99.40, 135.90) | 117.85(101.81, 139.10) | 120.88(103.71, 140.13) | 18.285 | ＜0.001 | **＜0.001** | 0.006 | 122.55(103.65, 140.31) | 122.49(105.04, 144.68) | 126.37(110.52, 146.48) | 19.580 | ＜0.001 | **＜0.001** | **0.007** |
| tyrosine/phenylalanine ratio | 1.80(1.46, 2.28) | 1.83(1.47, 2.34) | 1.80(1.43, 2.29) | 1.379 | 0.502 | 0.502 | - | 1.92(1.54, 2.42) | 1.88(1.49, 2.31) | 1.90(1.51, 2.36) | 3.015 | 0.221 | 0.288 | - |
| ornithine/citrulline ratio | 9.99(8.20, 12.28) | 9.92(8.02, 12.14) | 10.29(8.62, 12.64) | 17.286 | ＜0.001 | **＜0.001** | 0.005 | 9.85(7.98, 11.95) | 9.75(8.06, 11.90) | 10.22(8.40, 12.28) | 15.068 | 0.001 | **0.001** | **0.005** |
| C0 | 21.41(17.65, 25.65) | 20.75(17.18, 25.80) | 22.56(18.32, 27.15) | 31.928 | ＜0.001 | **＜0.001** | 0.010 | 21.07(17.64, 25.99) | 21.17(17.16, 25.61) | 22.21(18.46, 26.65) | 17.720 | ＜0.001 | **＜0.001** | **0.006** |
| C2 | 18.48(15.09, 22.39) | 17.97(14.58, 21.96) | 17.15(13.91, 20.61) | 30.339 | ＜0.001 | **＜0.001** | 0.009 | 18.29(14.87, 21.79) | 18.23(14.71, 22.17) | 17.25(14.06, 20.88) | 22.508 | ＜0.001 | **＜0.001** | **0.008** |
| C3 | 1.52(1.21, 1.90) | 1.49(1.16, 1.93) | 1.41(1.12, 1.82) | 19.396 | ＜0.001 | **＜0.001** | 0.006 | 1.48(1.18, 1.89) | 1.49(1.14, 1.91) | 1.43(1.14, 1.83) | 6.492 | 0.039 | 0.055 | - |
| C3DC + C4OH | 0.08(0.06, 0.10) | 0.08(0.06, 0.11) | 0.07(0.06, 0.09) | 57.217 | ＜0.001 | **＜0.001** | 0.018 | 0.08(0.06, 0.10) | 0.08(0.06, 0.11) | 0.07(0.06, 0.09) | 42.566 | ＜0.001 | **＜0.001** | **0.015** |
| C4 | 0.19(0.16, 0.23) | 0.19(0.16, 0.23) | 0.19(0.16, 0.23) | 1.083 | 0.582 | 0.582 | - | 0.20(0.16, 0.23) | 0.19(0.17, 0.23) | 0.20(0.17, 0.23) | 0.404 | 0.817 | 0.817 | - |
| C4DC + C5OH | 0.17(0.14, 0.20) | 0.17(0.14, 0.20) | 0.17(0.15, 0.20) | 2.156 | 0.340 | 0.377 | - | 0.17(0.14, 0.20) | 0.17(0.14, 0.20) | 0.17(0.14, 0.20) | 0.844 | 0.656 | 0.678 | - |
| C5 | 0.09(0.08, 0.12) | 0.09(0.08, 0.12) | 0.10(0.08, 0.12) | 13.762 | 0.001 | **0.002** | 0.004 | 0.10(0.08, 0.12) | 0.09(0.08, 0.11) | 0.10(0.08, 0.12) | 8.485 | 0.014 | **0.021** | **0.003** |
| C5:1 | 0.00(0.00,0.01) | 0.00(0.00,0.01) | 0.00(0.00,0.01) | 2.990 | 0.224 | 0.258 | - | 0.00(0.00,0.01) | 0.00(0.00,0.01) | 0.00(0.00,0.01) | 4.669 | 0.097 | 0.131 | - |
| C5DC + C6OH | 0.09(0.07, 0.11) | 0.09(0.07, 0.11) | 0.08(0.07, 0.10) | 20.334 | ＜0.001 | **＜0.001** | 0.006 | 0.09(0.07, 0.11) | 0.09(0.07, 0.11) | 0.09(0.07, 0.10) | 3.978 | 0.137 | 0.177 | - |
| C6 | 0.04(0.03, 0.04) | 0.03(0.03, 0.04) | 0.03(0.03, 0.04) | 11.523 | 0.003 | **0.004** | 0.004 | 0.04(0.03, 0.04) | 0.03(0.03, 0.04) | 0.03(0.03, 0.04) | 10.886 | 0.004 | **0.007** | **0.004** |
| C6DC | 0.10(0.08, 0.14) | 0.10(0.07, 0.13) | 0.12(0.09, 0.15) | 159.475 | ＜0.001 | **＜0.001** | 0.049 | 0.10(0.07, 0.14) | 0.09(0.07, 0.13) | 0.12(0.09, 0.15) | 182.969 | ＜0.001 | **＜0.001** | **0.065** |
| C8 | 0.04(0.03, 0.05) | 0.04(0.03, 0.05) | 0.04(0.03, 0.05) | 36.340 | ＜0.001 | **＜0.001** | 0.011 | 0.04(0.03, 0.05) | 0.04(0.03, 0.05) | 0.04(0.03, 0.05) | 13.030 | 0.001 | **0.003** | **0.005** |
| C8:1 | 0.10(0.08, 0.12) | 0.10(0.08, 0.12) | 0.10(0.08, 0.12) | 1.930 | 0.381 | 0.394 | - | 0.10(0.08, 0.12) | 0.10(0.07, 0.12) | 0.10(0.08, 0.12) | 2.268 | 0.322 | 0.369 | - |
| C10 | 0.05(0.04, 0.07) | 0.05(0.04, 0.07) | 0.05(0.04, 0.06) | 43.505 | ＜0.001 | **＜0.001** | 0.013 | 0.05(0.04, 0.07) | 0.05(0.04, 0.07) | 0.05(0.04, 0.06) | 14.573 | 0.001 | **0.001** | **0.005** |
| C10:1 | 0.06(0.04, 0.07) | 0.06(0.04, 0.07) | 0.06(0.04, 0.07) | 3.088 | 0.214 | 0.255 | - | 0.06(0.05, 0.07) | 0.06(0.04, 0.07) | 0.06(0.05, 0.07) | 1.600 | 0.449 | 0.498 | - |
| C10:2 | 0.01(0.01,0.01) | 0.01(0.01,0.01) | 0.01(0.01,0.01) | 37.795 | ＜0.001 | **＜0.001** | 0.012 | 0.01(0.01,0.01) | 0.01(0.01,0.01) | 0.01(0.01,0.01) | 20.655 | ＜0.001 | **＜0.001** | **0.007** |
| C12 | 0.06(0.05, 0.07) | 0.06(0.05, 0.07) | 0.05(0.04, 0.06) | 105.399 | ＜0.001 | **＜0.001** | 0.033 | 0.06(0.05, 0.07) | 0.06(0.05, 0.08) | 0.05(0.04, 0.07) | 48.043 | ＜0.001 | **＜0.001** | **0.017** |
| C12:1 | 0.04(0.03, 0.07) | 0.04(0.03, 0.06) | 0.04(0.03, 0.06) | 4.838 | 0.089 | 0.110 | - | 0.04(0.03, 0.07) | 0.04(0.03, 0.06) | 0.04(0.03, 0.07) | 0.877 | 0.645 | 0.678 | - |
| C14 | 0.16(0.13, 0.20) | 0.16(0.12, 0.20) | 0.15(0.12, 0.19) | 5.719 | 0.057 | 0.074 | - | 0.16(0.13, 0.19) | 0.16(0.13, 0.20) | 0.15(0.12, 0.19) | 3.778 | 0.151 | 0.188 | - |
| C14:1 | 0.07(0.06,0.09) | 0.07(0.05,0.09) | 0.07(0.05,0.08) | 22.099 | ＜0.001 | **＜0.001** | 0.007 | 0.07(0.05,0.09) | 0.07(0.06,0.09) | 0.07(0.05,0.08) | 11.289 | 0.004 | **0.006** | **0.004** |
| C14:2 | 0.02(0.01, 0.02) | 0.02(0.01, 0.02) | 0.02(0.01, 0.02) | 19.662 | ＜0.001 | **＜0.001** | 0.006 | 0.02(0.01, 0.02) | 0.02(0.01, 0.02) | 0.02(0.01, 0.02) | 8.974 | 0.011 | **0.017** | **0.003** |
| C14OH | 0.01(0.00,0.01) | 0.01(0.00,0.01) | 0.01(0.00,0.01) | 44.142 | ＜0.001 | **＜0.001** | 0.014 | 0.01(0.00,0.01) | 0.01(0.00,0.01) | 0.01(0.00,0.01) | 44.658 | ＜0.001 | **＜0.001** | **0.016** |
| C16 | 2.32(1.78, 2.95) | 2.34(1.79, 3.12) | 2.16(1.65, 2.76) | 34.727 | ＜0.001 | **＜0.001** | 0.011 | 2.28(1.75, 2.93) | 2.40(1.85, 3.05) | 2.13(1.64, 2.75) | 36.154 | ＜0.001 | **＜0.001** | **0.013** |
| C16:1 | 0.11(0.08, 0.15) | 0.11(0.08, 0.16) | 0.09(0.07, 0.13) | 79.827 | ＜0.001 | **＜0.001** | 0.025 | 0.11(0.08, 0.15) | 0.11(0.08, 0.15) | 0.10(0.07, 0.13) | 60.010 | ＜0.001 | **＜0.001** | **0.021** |
| C16:1OH | 0.03(0.02,0.04) | 0.03(0.02, 0.04) | 0.03(0.03,0.04) | 13.317 | 0.001 | **0.002** | 0.004 | 0.03(0.02,0.04) | 0.03(0.02,0.04) | 0.03(0.02,0.04) | 9.140 | 0.010 | **0.017** | **0.003** |
| C16OH | 0.01(0.01,0.01) | 0.01(0.01, 0.01) | 0.01(0.01,0.01) | 43.901 | ＜0.001 | **＜0.001** | 0.014 | 0.01(0.01, 0.01) | 0.01(0.01,0.01) | 0.01(0.01,0.01) | 23.978 | ＜0.001 | **＜0.001** | **0.008** |
| C18 | 0.72(0.59, 0.89) | 0.72(0.58, 0.91) | 0.69(0.56, 0.86) | 15.379 | ＜0.001 | **0.001** | 0.005 | 0.72(0.57, 0.87) | 0.74(0.59, 0.90) | 0.69(0.55, 0.86) | 19.813 | ＜0.001 | **＜0.001** | **0.007** |
| C18:1 | 1.31(1.09, 1.57) | 1.29(1.07, 1.57) | 1.24(1.02, 1.47) | 23.539 | ＜0.001 | **＜0.001** | 0.007 | 1.29(1.09, 1.56) | 1.31(1.11, 1.58) | 1.25(1.04, 1.48) | 18.333 | ＜0.001 | **＜0.001** | **0.006** |
| C18:1OH | 0.02(0.01,0.02) | 0.02(0.01,0.02) | 0.01(0.01,0.02) | 25.242 | ＜0.001 | **＜0.001** | 0.008 | 0.01(0.01,0.02) | 0.02(0.01,0.02) | 0.01(0.01,0.02) | 14.544 | 0.001 | **0.001** | **0.005** |
| C18:2 | 0.23(0.18, 0.30) | 0.23(0.17, 0.30) | 0.24(0.19, 0.30) | 2.079 | 0.354 | 0.378 | - | 0.23(0.18, 0.30) | 0.23(0.18, 0.29) | 0.24(0.18, 0.31) | 2.812 | 0.245 | 0.292 | - |
| C18OH | 0.01(0.00,0.01) | 0.01(0.00,0.01) | 0.01(0.00,0.01) | 29.823 | ＜0.001 | **＜0.001** | 0.009 | 0.01(0.00,0.01) | 0.01(0.00,0.01) | 0.01(0.00,0.01) | 24.961 | ＜0.001 | **＜0.001** | **0.009** |

Abbreviation: FDR: false discovery rate; Leu+Ile+Pro-OH: leucine+isoleucine+hydroxyproline, C0: free carnitine, C2: acetylcarnitine, C3: propionylcarnitine, C3DC + C4OH: malonylcarnitine + 3-hydroxybutyrylcarnitine, C4: butyrylcarnitine + isobutyrylcarnitine, C4DC + C5OH: methylmalonyl + 3-hydroxy-isovalerylcarnitine, C5: isovalerylcarnitine + methylbutyrylcarnitine, C5:1: tiglylcarnitine, C5DC + C6OH: glutarylcarnitine + 3-hydroxyhexanoylcarnitine, C6: hexanoylcarnitine, C6DC: methylglutarylcarnitine, C8: octanoylcarnitine, C8:1: octenoylcarnitine, C10: decanoylcarnitine, C10:1: decenoylcarnitine, C10:2: decadienoylcarnitine, C12: dodecanoylcarnitine, C12:1: dodecenoylcarnitine, C14: tetradecanoylcarnitine, C14:1: tetradecenoylcarnitine, C14:2: tetradecadienoylcarnitine, C14OH: 3-hydroxy-tetradecanoylcarnitine, C16: palmitoylcarnitine, C16:1: palmitoleylcarnitine, C16OH: 3-hydroxy-hexadecanoylcarnitine, C16:1OH: 3-hydroxy-hexadecenoylcarnitine, C18: stearoylcarnitine, C18:1: oleoylcarnitine, C18:1OH: 3-hydroxy-octadecenoylcarnitine, C18:2: linoleoylcarnitine, C18OH: 3-hydroxy-octadecanoylcarnitine.

Alt text: Table presenting sex-stratified analyses of peripheral blood amino acid, acylcarnitine levels, and selected amino acid ratios in neonates born to three maternal groups (overt hypothyroidism during pregnancy, subclinical hypothyroidism during pregnancy, control), with separate data for male and female neonates (all expressed as median (25th, 75th percentile)); presents Kruskal-Wallis H statistic, P value, false discovery rate (FDR), and epsilon‑squared (ε²; reported only for statistically‑significant comparisons) effect-size values for intergroup statistical differences in each sex subgroup.
