# Supplementary material for: Overt and subclinical hypothyroidism during pregnancy: effects on neonatal amino acids, acylcarnitines, and TSH levels
Source: Front Endocrinol (Lausanne). 2026 Sep 25;17:1872893. doi: 10.3389/fendo.2026.1872893 (PMC13649493; doi:10.3389/fendo.2026.1872893)
Supplement: Supplementary file 6 [file Table6.docx]

**T****able** **S6 Associations of overt and subclinical hypothyroidism during pregnancy with neonatal peripheral blood acylcarnitine levels (with the control group as reference).**

| **Acylcarnitine** | **Overt hypothyroidism during pregnancy group**  **(N=1987)** | | | **Subclinical hypothyroidism during pregnancy group**  **`(N=2023)** | | |
| --- | --- | --- | --- | --- | --- | --- |
|  | **Std. β** | **β(95%CI）** | **FDR*** | **Std. β** | **β(95%CI）** | **FDR*** |
| C0 | -0.054 | -0.782(-1.191 to -0.372) | **＜0.001** | -0.075 | -1.076(-1.482 to -0.670) | **＜0.001** |
| C2 | 0.087 | 1.073(0.718 to 1.428) | **＜0.001** | 0.088 | 1.091(0.739 to 1.442) | **＜0.001** |
| C3 | 0.048 | 0.062(0.025 to 0.099) | **0.002** | 0.051 | 0.065(0.029 to 0.101) | **0.001** |
| C3DC + C4OH | 0.082 | 0.008(0.005 to 0.011) | **＜0.001** | 0.108 | 0.010(0.007 to 0.013) | **＜0.001** |
| C4 | - | 0.000(-0.004 to 0.004) | 0.982 | - | 0.000(-0.004 to 0.003) | 0.961 |
| C4DC + C5OH | - | 0.000(-0.006 to 0.007) | 0.973 | - | 0.003(-0.003 to 0.010) | 0.391 |
| C5 | -0.059 | -0.005(-0.007 to -0.003) | **＜0.001** | -0.055 | -0.005(-0.007 to -0.002) | **＜0.001** |
| C5:1 | - | 0.000(0.000 to 0.001) | 0.270 | - | 0.000(0.000 to 0.000) | 0.992 |
| C5DC + C6OH | 0.049 | 0.003(0.001 to 0.004) | **0.001** | 0.044 | 0.002(0.001 to 0.004) | **0.004** |
| C6 | 0.065 | 0.002(0.001 to 0.003) | **＜0.001** | 0.056 | 0.001(0.001 to 0.002) | **＜0.001** |
| C6DC | -0.156 | -0.017(-0.020 to -0.014) | **＜0.001** | -0.224 | -0.024(-0.028 to -0.021) | **＜0.001** |
| C8 | 0.072 | 0.003(0.002 to 0.004) | **＜0.001** | 0.068 | 0.002(0.001 to 0.003) | **＜0.001** |
| C8:1 | - | 0.002(-0.001 to 0.004) | 0.181 | - | 0.001(-0.001 to 0.003) | 0.353 |
| C10 | 0.067 | 0.004(0.002 to 0.005) | **＜0.001** | 0.087 | 0.005(0.003 to 0.006) | **＜0.001** |
| C10:1 | - | 0.001(-0.001 to 0.002) | 0.308 | - | 0.000(-0.001 to 0.001) | 0.729 |
| C10:2 | 0.072 | 0.001(0.000 to 0.001) | **＜0.001** | 0.069 | 0.001(0.000 to 0.001) | **＜0.001** |
| C12 | 0.103 | 0.006(0.004 to 0.008) | **＜0.001** | 0.144 | 0.009(0.007 to 0.010) | **＜0.001** |
| C12:1 | - | 0.000(-0.001 to 0.002) | 0.841 | - | -0.001(-0.003 to 0.001) | 0.297 |
| C14 | - | 0.002(-0.001 to 0.006) | 0.216 | 0.037 | 0.004(0.001 to 0.007) | **0.016** |
| C14:1 | 0.060 | 0.004(0.002 to 0.005) | **＜0.001** | 0.062 | 0.004(0.002 to 0.005) | **＜0.001** |
| C14:2 | 0.057 | 0.001(0.000 to 0.001) | **<0.001** | 0.047 | 0.001(0.000 to 0.001) | **0.002** |
| C14OH | 0.094 | 0.001(0.001 to 0.001) | **＜0.001** | 0.117 | 0.001(0.001 to 0.002) | **＜0.001** |
| C16 | 0.069 | 0.139(0.082 to 0.196) | **＜0.001** | 0.117 | 0.235(0.179 to 0.292) | **＜0.001** |
| C16:1 | 0.110 | 0.013(0.010 to 0.017) | **＜0.001** | 0.158 | 0.019(0.016 to 0.022) | **＜0.001** |
| C16:1OH | -0.050 | -0.001(-0.002 to -0.001) | **0.001** | -0.060 | -0.002(-0.002 to -0.001) | **＜0.001** |
| C16OH | 0.069 | 0.001(0.000 to 0.001) | **＜0.001** | 0.113 | 0.001(0.001 to 0.001) | **＜0.001** |
| C18 | 0.040 | 0.021(0.006 to 0.036) | **0.008** | 0.079 | 0.042(0.027 to 0.057) | **＜0.001** |
| C18:1 | 0.074 | 0.060(0.036 to 0.083) | **＜0.001** | 0.090 | 0.073(0.050 to 0.096) | **＜0.001** |
| C18:1OH | 0.047 | 0.001(0.000 to 0.001) | **0.002** | 0.093 | 0.001(0.001 to 0.002) | **＜0.001** |
| C18:2 | - | 0.001(-0.005 to 0.007) | 0.839 | - | -0.001(-0.007 to 0.004) | 0.729 |
| C18OH | 0.070 | 0.001(0.000 to 0.001) | **＜0.001** | 0.099 | 0.001(0.001 to 0.001) | **＜0.001** |

Abbreviation: FDR, false discovery rate; C0, free carnitine; C2, acetylcarnitine; C3, propionylcarnitine; C3DC + C4OH, malonylcarnitine + 3-hydroxybutyrylcarnitine; C4, butyrylcarnitine + isobutyrylcarnitine; C4DC + C5OH, methylmalonyl + 3-hydroxy-isovalerylcarnitine; C5, isovalerylcarnitine + methylbutyrylcarnitine; C5:1, tiglylcarnitine; C5DC + C6OH, glutarylcarnitine + 3-hydroxyhexanoylcarnitine; C6, hexanoylcarnitine; C6DC, methylglutarylcarnitine; C8, octanoylcarnitine; C8:1, octenoylcarnitine; C10, decanoylcarnitine; C10:1, decenoylcarnitine; C10:2, decadienoylcarnitine; C12, dodecanoylcarnitine; C12:1, dodecenoylcarnitine; C14, tetradecanoylcarnitine; C14:1, tetradecenoylcarnitine; C14:2, tetradecadienoylcarnitine; C14OH, 3-hydroxy-tetradecanoylcarnitine; C16, palmitoylcarnitine; C16:1, palmitoleylcarnitine; C16:1OH, 3-hydroxy-hexadecenoylcarnitine; C16OH, 3-hydroxy-hexadecanoylcarnitine; C18, stearoylcarnitine; C18:1, oleoylcarnitine; C18:1OH, 3-hydroxy-octadecenoylcarnitine; C18:2, linoleoylcarnitine; C18OH, 3-hydroxy-octadecanoylcarnitine.

The models were adjusted for gestational age at delivery, birth weight, neonatal gender and age at heel prick. *FDR was calculated to account for multiple comparisons using the Benjamini-Hochberg method.

Alt text: Table presenting regression analyses of associations between overt and subclinical hypothyroidism during pregnancy and neonatal peripheral blood acylcarnitine levels (with the control group as reference); shows standardized β (Std. β; reported only for statistically‑significant comparisons), β coefficients (95% confidence interval, 95%CI) and false discovery rate (FDR) for the two hypothyroidism groups separately, with all analytical models adjusted for gestational age at delivery, birth weight, neonatal gender and age at heel prick.
